# Implant Evaluation of an Insertable Cardiac Monitor Outside the Electrophysiology Lab Setting

**DOI:** 10.1371/journal.pone.0071544

**Published:** 2013-08-15

**Authors:** Roman Pachulski, James Cockrell, Hemant Solomon, Fang Yang, John Rogers

**Affiliations:** 1 South Texas Heartbeat, San Antonio, Texas, United States of America; 2 Cardiovascular Consultants PA, Takoma Park, Maryland, United States of America; 3 Southeastern Heart and Vascular Center, Greensboro, North Carolina, United States of America; 4 Medtronic, Inc., CRDM Clinical Research, Minneapolis Minnesota, United States of America; 5 Scripps Clinic, La Jolla, California, United States of America; Sapienza University of Rome, Italy

## Abstract

**Background:**

To date, insertable cardiac monitors (ICM) have been implanted in the hospital without critical evaluation of other potential settings. Providing alternatives to in-hospital insertion may increase access to ICM, decrease waiting times for patients awaiting diagnosis, and reduce hospital resources.

**Methods:**

This was a prospective, non-randomized, clinical trial involving nine clinical sites throughout the United States designed to assess the feasibility of ICM implants in a non-hospital setting. Other than the Reveal® ICM, implant supplies and techniques were left to physician discretion in patients who met indications. Patients were followed up to 90 days post-implant. The primary objective was to characterize the number of procedure-related adverse events that required surgical intervention within 90 days.

**Results:**

Sixty-five patients were implanted at nine out-of-hospital sites. The insertion procedure was well tolerated by all patients. There were no deaths, systemic infections or endocarditis. There were two (3%) procedure-related adverse events requiring device explant and four (6%) adverse events not requiring explant. ICM use led to 16 diagnoses (24.6%) with 9 patients proceeding to alternate cardiac device implants during the course of the 90-day follow up.

**Conclusion:**

Out-of-hospital ICM insertion can be accomplished with comparable procedural safety and represents a reasonable alternative to the in-hospital setting.

**Clinicaltrials.gov registration number:** NCT01168427

## Introduction

Subcutaneous insertable cardiac monitors (ICM) are indicated for use in patients with clinical syndromes or symptoms suggestive of cardiac arrhythmias [1]–[5]. To date, ICM implantation has been limited to the in-hospital setting, utilizing resources historically reserved for more complex cardiac devices that require transvenous leads and use of fluoroscopy. Whether alternatives to in-hospital ICM implants exist has not been previously explored.

Movement of minor surgical procedures to a less resource-intensive, in-office setting has been adopted by multiple clinical specialties including vascular and plastic surgery, gynecology, and urology [6]–[9]. The major concern with moving a procedure out of the hospital setting is patient safety. Overall, in-hospital ICM implant complication rates are relatively low [10]–[12] and are less than pacemaker implants [13]. Infection rates for in-hospital ICM implants range from 2.3–4.3% [10]–[12]. The ICM is a fully functional, self-contained diagnostic device which is inserted subcutaneously. Unlike pacemakers and implantable cardioverter defibrillators, ICM do not require central vascular access or direct contact with the endocardium. Consequently, ICM do not carry the same risk of endocardial infection [14], [15]. The ICM is typically inserted with a 1–2 cm incision and does not require conscious sedation [16], [17]. Thus, if the characteristics of the new environment are carefully considered and sterile technique is adhered to, the ICM may be an ideal candidate for successful insertion in the office or clinic setting.

The purpose of this study was to document the feasibility of out-of-hospital insertion of the Medtronic Reveal® (Minneapolis, MN) ICM outside of the hospital environment. The primary objective was to document all procedure-related adverse events that required surgical intervention within 90 days of device placement. Secondary objectives included documenting all other procedure-related adverse events, time and resource utilization, insertion technique, and device functionality.

## Methods

The protocol for this trial and supporting CONSORT checklist are available as supporting information; see Checklist S1 and Protocol S1.

### Ethics Statement

The study protocol and informed consent was approved by the Western Institutional Review Board, Olympia WA for 8 of the 9 participating institutions and by the local Scripps IRB, La Jolla, CA for the Scripps Clinic at Torrey Pines. All patients were provided with and signed the IRB approved written informed consent.

### Study Overview

This was a prospective, non-randomized, clinical trial (ClinicalTrials.gov NCT01168427) involving experienced implanters at nine clinic sites in the United States. The Medtronic Reveal® DX or Reveal® XT ICM was implanted in adult, non-pregnant subjects with established clinical indications using techniques and procedures similar to those for in-hospital insertion. Patients were screened and eligible patients enrolled as they presented to the practices of the study physicians. Patients with limited life expectancy (<12 months), active infection (within previous 30 days) or otherwise at high risk for infection, and those with unusual thoracic anatomy or scarring at the implant site were excluded from the study.

### Study Procedures

The insertion procedure was required to take place in a setting other than an operating room or electrophysiology laboratory. The room utilized for the procedure was left to physician discretion. The Reveal® DX or the Reveal® XT device and the 2090 CareLink® Programmer were the only required components for this study. Physicians had access to disposable items similar to those used for in-hospital insertion (syringes, scalpels, gowns, sterile drapes, dressings, etc.) with specific supplies utilized at the discretion of the study investigator. The type and route of administration of perioperative prophylactic antibiotics were also left to individual physician practices. All centers were instructed to adhere to infection control procedures and room preparation guidelines. Patient sedation was limited to oral anxiolytics and analgesics at the physician’s discretion. In-office follow-up visits occurred at 30 and 90 days post-implant to identify any adverse events that could be attributable to the implant procedure. If necessary, the 90-day visit could be conducted by telephone.

### Data Analysis

All complications were documented throughout the study. An Adverse Event Adjudication Committee (AEAC) comprised of four independent, non-participating physicians adjudicated each event and classified it as procedure-related, infection-related, serious or not serious, and whether the event was a complication. Procedure-related adverse events were defined as events due to procedures related to the implantation or surgical modification of the device. Complications were defined as adverse events that resulted in death, involved any termination of significant device function, or required invasive intervention. Serious complications were defined as events leading to death or serious deterioration of the health of the subject. The primary analysis used the Kaplan-Meier method to estimate the event rate and associated upper bound of 95% two-sided confidence interval at 90 days post-implant. For this analysis, days of follow-up were computed as the days from implant to the onset date of the first event for subjects with events. For subjects without an event, days of follow-up were calculated as days since implant to last follow-up date. The last follow-up date was defined as the date of the 90-day visit, death, or exit, whichever came first. A simulation study using Kaplan-Meier and Exact binomial method was conducted to determine the sample size of 65 patients, assuming that 30 day and 90 day event rate were 4% and 4.5% [12]. Summary statistics were calculated and analyses performed using SAS statistical software (SAS Institute Inc., Cary, NC).

## Results

Baseline characteristics of the study patients are listed in Table 1. Sixty-six patients were enrolled at nine centers. The first patient was enrolled on August 13, 2010 and the last patient enrolled on January 10, 2010. Thirty-six (54.5%) of the enrolled patients were male with mean age 60±18 years old. The majority (70.0%) of patients had no prior cardiovascular procedural therapy while 19 (28.8%) had a history of carotid or coronary revascularization. Sixty-five devices were inserted with 55 (85%) for presyncope or palpitations defying diagnosis and 10 (15%) to monitor AF burden. One patient was enrolled but developed pneumonia prior to device implantation and was exited from the study. Of the implanted devices, 12 (18%) were Reveal® DX and 53 (82%) were the Reveal® XT model which includes atrial fibrillation capabilities.

**Table 1:** Patient Baseline Demographics.

		Total (N = 66)
Age		59.9±18 (range 19–92)
Male sex		36 (54.5%)
Race/Ethnic:	White or Caucasian	59 (89.4%)
	Black or African American	6 (9.1%)
	Hispanic or Latino	1 (1.5%)
Weight (kg)		88.8±24.2 (range 46.3–170)
BMI		30.3±7.8 (range 17.2–60.7)
Hypertension		43 (65.2%)
Coronary artery disease		22 (33.3%)
Congestive heart failure		5 (7.6%)
Diabetes mellitus		15 (22.7%)
History of syncope		37 (56.1%)
History of atrial fibrillation		17 (25.8%)

Fifteen physicians with extensive prior ICM experience performed the implants and included five cardiologists and ten electrophysiologists. Of the 15 implanting physicians, 9 placed 2–5 devices, representing 34 insertions; 3 implanters placed 9–10 devices for 28 procedures; and 3 implanters placed one ICM each.

**Figure 1:**
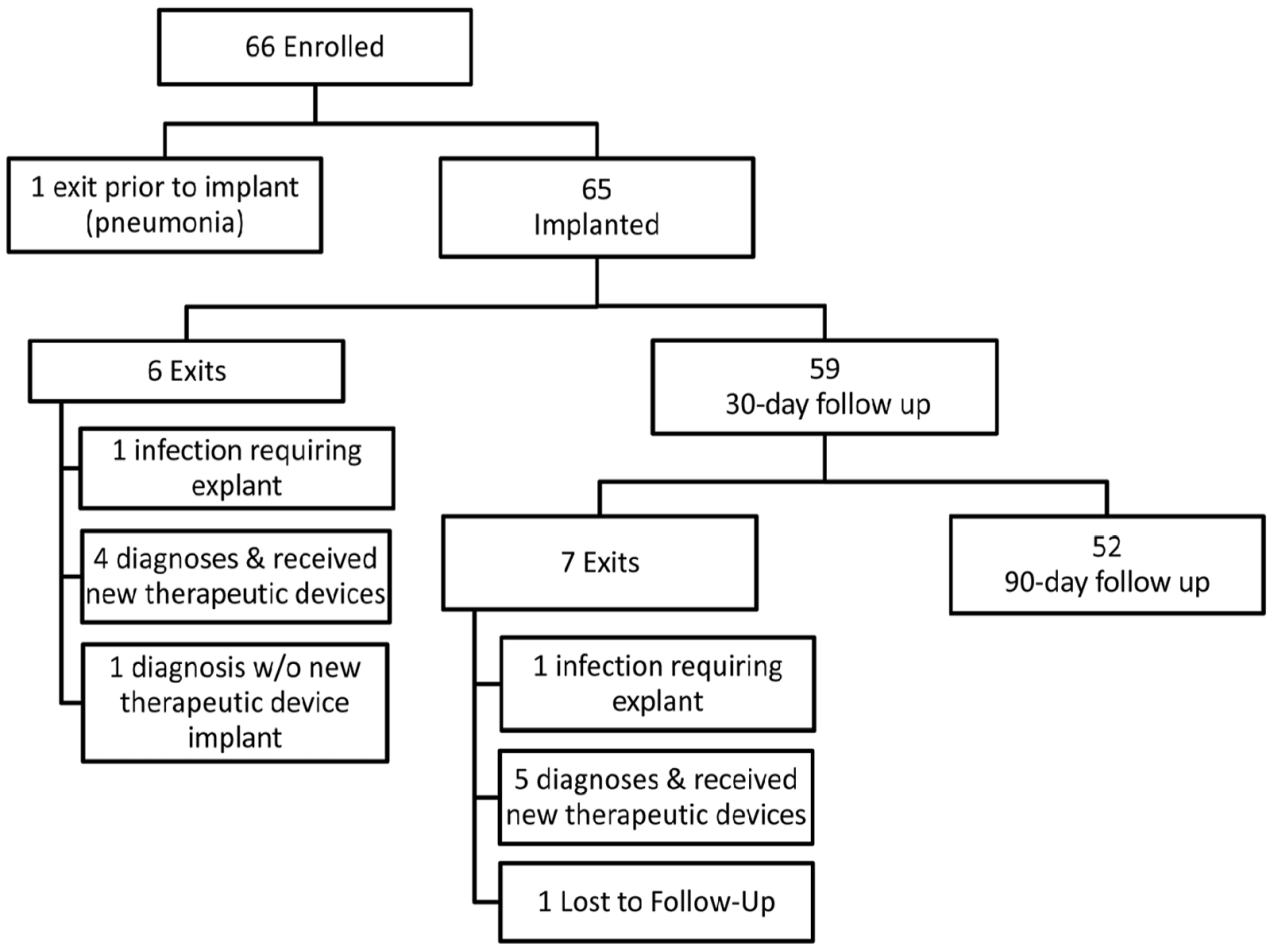
Patient Flow Chart: Enrolled patient outcomes.

Fifty-nine patients (89%) completed the 30-day follow up (Figure 1) and 52 (80%) completed the entire 90-day follow up period. Of the 14 patients exited early, 1 patient was withdrawn from the study prior to ICM placement (pneumonia). Six were exited prior to 30-day follow up (5 diagnoses obtained, 1 infection) and 7 exited between 30 and 90 days (5 diagnosis obtained, 1 infection, 1 lost to follow-up). A total of 16 (25%) patients received a diagnosis during the 90 days of follow-up based on the ICM data (mean time to diagnosis 37 days; range 3 to 92 days) including six patients who received a pacemaker or implantable cardioverter-defibrillator. The last patient completed follow up and exited the study on April 13, 2011.

Overall, the out-of-hospital implants were well tolerated by patients. Of the 65 implanted patients, there were 2 (3%) procedure-related adverse events which required resolution by surgical intervention within 90 days of the implant procedure. There were four (6%) additional adverse events that were characterized as not serious by the AEAC and did not require explant. No patient required parenteral antibiotics for infection-related adverse events. There were no deaths, systemic infections or cases of endocarditis.

The two procedure-related adverse events leading to surgical intervention and subsequent explantation of the device were adjudicated by the AEAC as implant site infections. One of these events occurred on day 25 in a 37 year old with no co-morbidities and was preceded by a vesicular erythematous rash immediately under the occlusive dressing tape. This patient was found to have a family history of tape allergy. The second infection-related event requiring explant occurred on day 53 in a 73 year old with diabetes and hypertension. It was preceded by pain and erythema and treated with oral antibiotics prior to explant. Both patients received prophylactic oral antibiotics at appropriate intervals prior to implant. The pocket was not flushed with antibiotic-containing solutions in either procedure. Physicians in both cases wore sterile gowns, gloves and masks during the implant and did not report problems with the procedure. Based on these events, the Kaplan-Meier estimate of the primary event rate requiring surgical intervention at 90 days post implant was 3.4% with a 95% confidence interval upper bound at 12.9%. Both events resolved upon device explantation.

Of the four minor events, one was a scalp rash attributed to prophylactic vancomycin infusion at the time of implant (treated with diphenhydramine); one minor dressing allergy manifested as a self-limiting rash, and two were superficial infection events that resolved with oral antibiotic therapy. Both patients with superficial infections received prophylactic antibiotics; one intravenously (IV) and one orally. Both ICM pockets were flushed with antibiotic-containing solutions during the procedure. One patient had a history of Type-2 diabetes. The four, non-primary events all resolved without surgical intervention and all occurred within 12 days of the procedure.

All 65 (100%) of patients received preoperative antibiotics generally targeting gram-positive cocci with 35 patients (54%) receiving oral antibiotics and 30 patients (46%) receiving IV antibiotics. The most common oral and IV antibiotics were cephalexin and cefazolin, respectively. Forty patients (65.5%) had the pocket flushed with an antibiotic-containing solution. A majority (61.5%) of patients received only preoperative antibiotics with the remaining 25 patients receiving some form of oral, postoperative antibiotics for varying durations.

The patient and physician preparation as well as the type of room used for implant procedure was left up to the discretion of the investigator (Table 2). The majority of the procedures (43; 66%) were performed at 6 centers in standard clinical examination room. The remaining 22 (34%) procedures were performed at centers in utilizing a TEE room, vein ablation room and a cardiac diagnostic lab not associated with a hospital (Table 2). Four (44%) of the procedure rooms had air flow control (either filter or positive pressure) and 7 had a sink present in the room.

**Table 2:** Implant Preparation and Technique.

		Total (N = 65)
**Type of room**		
	Examination room	43 (66.1%)
	Outpatient diagnostic laboratory	10 (15.4%)
	Vein ablation room	6 (9.2%)
	TEE* room	6 (9.2%)
**Patient preparation**		
	Topical disinfectant	65 (100%)
	Sterile drape	65 (100%)
	Mask	31 (47.7%)
	Subcutaneous analgesics	65 (100%)
	Oral benzodiazepines	40 (62%)
	Oral analgesics	13 (20%)
**Physician preparation**		
	Sterile gloves	65 (100%)
	Mask	65 (100%)
	Sterile gown	63 (97%)
	Wet scrub	55 (84.6%)
	Dry scrub	10 (15.4%)
**Hemostasis obtained** †		
	Cautery	53 (81.5%)
	Gauze	41 (63.1%)
	Suture	8 (12.3%)
	Manual pressure	5 (7.7%)
	Clamp	1 (1.5%)
**Device anchored with sutures**		
	No sutures	14 (21.5%)
	1 suture	23 (35.4%)
	2 suture	28 (43.1%)
**Number of layers for wound closure**		
	1	3 (4.6%)
	2	42 (64.6%)
	3	20 (30.8%)

* TEE: transesophageal echocardiogram room;

† multiple methods could be employed.

The overall procedure time was 118±51 (range: 52–238) minutes from the start of the procedure preparation until the end of subject recovery, with a mean “skin to skin” time of 26±10 (range: 8–56) minutes. The implant procedure typically required one physician and one registered nurse. Most patients (72%) were prepped in the procedure room. Physicians prepped in the implant room in 43 cases (66%). The device was inserted above the fourth rib in 60 (92%) cases and ICM orientation was vertical in 28 (43%) and horizontal in 13 (20%) with the remainder at various angles.

## Discussion

This study allowed physicians to insert an ICM in indicated subjects utilizing their own techniques outside of the hospital operating room or cardiac catheterization lab. The major finding of this trial of 65 patients who had ICM implants outside of the traditional hospital setting was that this approach is indeed feasible with 91% of patients having no adverse events. Only 2 patients exhibited complications within 90 days requiring surgical intervention and device explantation. Four patients had AEAC-adjudicated minor adverse events. Despite the variation in techniques employed, this study demonstrated that with careful selection of an out-of-hospital environment, ICM insertion can be performed with comparable complication rates to the in-hospital setting.

There has been gradual evolution in the procedure-related aspects of cardiac implantable electronic devices such as pacemakers and defibrillators. These devices were initially implanted in surgical suites by surgeons to minimize bacterial contamination and infection. More recently, complication rates for in-hospital cardiac catheterization or electrophysiology laboratory implantation were found to be comparable [18]–[20]. With the advent of the ICM in the late 1990s, physicians adopted a similar in-hospital implant approach despite the fact that these devices are leadless and do not require transvenous access. This minimally invasive procedure of ICM placement does not require the resources found in hospital cardiac catherization or electrophysiology labs, and movement of this procedure to a safe, out of hospital setting frees up these facilities for more complex cardiac procedures.

The concept of moving a surgical procedure to a less resource-intensive setting is not new [6]–[9]. Most of these migrations were driven in part by advances in operative technique, technology, and anesthesia as well as changes in procedural economics and patient acceptance. Such changes in procedural site of service are usually accompanied by valid concerns as to the level of patient and procedure-related safety in the new setting.

The most costly adverse event that can occur as a result of an ICM implant, in both resources and patient health, is surgical intervention. Because of this, the study focused on these events as a primary objective. The Kaplan-Meier estimate of rate of adverse events requiring surgical intervention within 90 days was 3.4% and was comparable to previously reported in-hospital ICM insertion complication rates [10]–[12]. This study is the first to prospectively document the implantation experience and associated adverse events as primary outcomes for an ICM.

In this study, all patients received preoperative antibiotic prophylaxis in light of previous data showing beneficial results from randomized, double-blind, placebo-controlled trials for other implantable cardiac devices [18]. Furthermore, we believe that preoperative antibiotic prophylaxis reflect the current practice for in-hospital ICM insertions. Four patients (6.1%) experienced infectious complications during the study; two were events requiring explant and two were superficial and treated with oral courses of antibiotics. This percentage is slightly higher than the infection rate reported for in-hospital ICM insertion (2.3–4.3%) [10]–[12]. It is important to note that these prior publications may have underestimated the rate of infection-related adverse events because they were not designed to identify adverse events as a primary endpoint. Therefore, it is difficult to directly compare our results with these historical values. There are a number of potential reasons for the four infections. Two of the procedure-related adverse events (one requiring explant) occurred in diabetic patients; a population known to have higher rates of post-operative infections [21]. In one case requiring device explant, the patient developed a rash in the area of the occlusive dressing that may have been an allergic reaction to the dressing. Whether this resulted in an environment conducive for infection is unknown. Although both patients with superficial infections received antibiotic prophylaxis, one received oral doxycycline almost 12 hours prior to the implant start time, possibly rendering it ineffective. In the other case, cefazolin was administered just prior to the start of the implant. No correlation could be found for other factors such as site preparation, hemostasis, wet versus dry scrub, in-room versus out-of-room physician prep, or number of closure layers due to the small numbers involved.

Two separate adverse events were recorded as allergic reactions to the occlusive dressing. Allergic reactions due to dressings have been previously reported [22]. In view of our findings, caution should be employed when selecting wound dressings for patients.

### Study Limitations

This study was designed to assess the feasibility of out-of-hospital ICM implants and therefore patient preparation and physician practices were not strictly controlled. In 15% of the cases, the procedure room was similar to the hospital setting (physician owned outpatient diagnostic laboratory). This was a laboratory distinctly separate from the hospital and had not previously been used to implant cardiac devices. Nonetheless, it reflects a different environment than the other out-of-hospital locations. Only 52 (80%) of the 65 patients were followed out to the planned 90 day observation period. As identified in Fig. 1, the majority of these cases were due to the identification of a diagnosis using information from the ICM. Finally, suspected infections were not confirmed by laboratory culture and the diagnosis was made by the individual physician with eventual adjudication by the AEAC.

## Conclusion

The results of this trial in a non-structured, out-of-hospital setting demonstrate that the ICM can be inserted with comparable patient outcomes to in-hospital insertion. More study is needed to clarify potential gains in associated cost savings, workflow, and the utility of a structured implant protocol.

## Supporting Information

Checklist S1 **CONSORT checklist.**
(DOC)

Protocol S1 **Trial protocol.**
(PDF)
