# Supplementary material for: Implant Evaluation of an Insertable Cardiac Monitor Outside the Electrophysiology Lab Setting
Source: PLoS One. 2013 Aug 15;8(8):e71544. doi: 10.1371/journal.pone.0071544 (PMC3744555; doi:10.1371/journal.pone.0071544)
Supplement: Protocol S1 — Trial protocol. (PDF) [file pone.0071544.s002.pdf]

Reveal In-Office Implants

Clinical Investigation Plan

Version 1
10 May 2010

Medtronic, Inc.
8200 Coral Sea Street NE
Mounds View, MN U.S.A. 55112
1-800-328-2518

CONTACT INFORMATION

Medtronic contact information is provided below. This information is subject to change during the course of the clinical study. Periodic updates to study contact information will be sent to the centers as necessary.

SPONSOR

Study-Specific Contact:
Steve Erickson<br>Principal Clinical Research Specialist<br>Direct Phone: +1-763-526-2759<br>Direct Fax: +1-763-479-9321<br>steve.a.erickson@medtronic.com

Table of Contents

CONTACT INFORMATION 2

1 INTRODUCTION 6

1.1 STUDY PURPOSE 6
1.2 STUDY SCOPE 6

2 BACKGROUND AND JUSTIFICATION 6

3 SYSTEM DESCRIPTION AND INTENDED USE 8

3.1 REVEAL DX OR REVEAL XT INSERTABLE CARDIAC MONITOR 8
3.2 MEDTRONIC MODEL 2090 CARELINK® PROGRAMMER 8
3.3 REVEAL PATIENT ASSISTANT DEVICE 9
3.4 INTENDED USE 9
3.5 REGULATORY CLASSIFICATION 9

4 METHODOLOGY 10

4.1 STUDY OBJECTIVES 10
4.2 SUBJECT SELECTION 10
4.3 MINIMIZATION OF BIAS 11

5 STUDY PROCEDURES 12

5.1 INFORMED CONSENT PROCESS 12
5.2 DATA COLLECTION 14
5.3 SUBJECT FOLLOW-UP SCHEDULE 16
5.4 ENROLLMENT 16
5.5 BASELINE 16
5.6 IMPLANT 16
5.7 30-DAY FOLLOW-UP VISIT 17
5.8 90-DAY FOLLOW-UP VISIT 17
5.9 UNSCHEDULED VISIT 17
5.10 ADVERSE EVENT 18
5.11 SYSTEM MODIFICATION 18
5.12 TECHNICAL OBSERVATION 18
5.13 STUDY EXIT 18
5.14 MEDICATIONS 19
5.15 EQUIPMENT UTILIZATION SURVEY 20

6 DEVICE STORAGE, HANDLING AND TRACEABILITY 20

7 STUDY DEVIATIONS 20

8 ADVERSE EVENTS 21

8.1 ADVERSE EVENT COLLECTION REQUIREMENTS AND DEFINITIONS 22
8.2 ADVERSE EVENT CLASSIFICATION AND REPORTING 24
8.3 DEATH CLASSIFICATIONS AND REPORTING REQUIREMENTS 25
8.4 ADVERSE EVENT ADVISORY COMMITTEE REVIEW (AEAC) 27
8.5 MEDICAL DEVICE REPORTING (MDR) REQUIREMENTS FOR USER FACILITIES 27
8.6 INVESTIGATOR ADVERSE EVENT RECORDS AND REPORTING REQUIREMENTS 28

9 RISK ANALYSIS....28

9.1 RISK MINIMIZATION....29
9.2 POTENTIAL BENEFITS....29

10 PLANNED STUDY CLOSURE, EARLY TERMINATION OF STUDY OR SUSPENSION....29

10.1 PLANNED STUDY CLOSURE....29
10.2 EARLY TERMINATION OR SUSPENSION....29

11 STATISTICAL METHODS AND DATA ANALYSIS....31

11.1 PRIMARY OBJECTIVES....31
11.2 SECONDARY OBJECTIVES....33

12 DATA AND QUALITY MANAGEMENT....34

13 WARRANTY INFORMATION....35

14 MONITORING....35

14.1 MONITORING VISITS....35

15 REQUIRED RECORDS AND REPORTS....35

15.1 INVESTIGATOR RECORDS....35
15.2 INVESTIGATOR REPORTS....36
15.3 SPONSOR RECORDS....37
15.4 SPONSOR REPORTS....37

APPENDICES....39

APPENDIX A: CASE REPORT FORMS....40
APPENDIX B: PRELIMINARY PUBLICATION PLAN....68
APPENDIX C: DATA MONITORING COMMITTEE (DMC)....70
APPENDIX D: STUDY OVERVIEW....71
APPENDIX E: INFORMED CONSENT TEMPLATE....72
APPENDIX F: PARTICIPATING INVESTIGATORS AND INSTITUTIONS....85
APPENDIX G: IRB LIST....86
APPENDIX H: LABELING....87
APPENDIX I: BIBLIOGRAPHY....88

TABLES

TABLE 1: DATA COLLECTION AND STUDY PROCEDURE REQUIREMENTS AT SUBJECT VISITS 14
TABLE 2: STUDY VISIT WINDOWS 16
TABLE 3: ADVERSE EVENT DEFINITIONS 22
TABLE 4: ADVERSE EVENT RESPONSIBILITIES 25
TABLE 5: DEATH CLASSIFICATION DEFINITIONS 26
TABLE 6: SUBJECT DEATH CLASSIFICATION RESPONSIBILITIES 27
TABLE 7: INVESTIGATOR ADVERSE EVENT REPORTS 28
TABLE 8: 90-DAY EVENT RATES 32
TABLE 9: INVESTIGATOR REPORTING REQUIREMENTS 37
TABLE 10: SPONSOR REPORTS 38

ABBREVIATIONS

AE Adverse Event
AEAC Adverse Event Advisory Committee
AF Atrial Fibrilation
AT Atrial Tachycardia
CIP Clinical Investigation Plan
CRF Case Report Form
CRT Cardiac Resynchronization Therapy
CTA Clinical Trial Agreement
DMC Data Monitoring Committee
ECG Electrocardiogram
EP Electrophysiology
e-CRF Electronic Case Report Form
FDA Food and Drug Administration
FDAA Food and Drug Administration Amendments Act
FU Follow-Up
HIPAA Health Insurance Privacy and Accountability Act
ICD Implantable Cardioverter Defibrillator
ICM Insertable Cardiac Monitor
IDE Investigational Device Exemption
IPG Implantable Pulse Generator
IRB Institutional Review Board
ISO International Organization for Standardization
MedDRA Medical Dictionary for Regulatory Activities
PC Publication Committee
RDC Remote Data Capture
SAE Serious Adverse Event
SOP Standard Operating Procedure
S2D Save-To-Disk
US United States
VT Ventricular Tachycardia

1 Introduction

1.1 Study Purpose

Medtronic, Inc. is sponsoring the Reveal® In-Office Implants study, a prospective, non-randomized, multi-center post-market clinical trial.
The purpose of this study is to gather evidence about the safety and feasibility of performing the implant procedure for the Reveal Insertable Cardiac Monitor (ICM) in an office or clinic setting rather than the traditional hospital operating room, cardiac catherization or electrophysiology (EP) laboratory setting. Data collected in this study may be published and potentially utilized in conjunction with submission to reimbursement agencies in support of seeking an appropriate mechanism for reimbursement of the practice expenses (device implant, procedure-related supplies, patient care staff, facility expenses, etc) associated with a "non-facility" site of service.

1.2 Study Scope

The Reveal In-Office Implants study will be conducted at approximately 12 centers located in the United States.
The study is expected to enroll approximately 65 subjects who will all be implanted with a Medtronic market released Reveal® DX or Reveal® XT ICM.

To satisfy enrollment for the objectives, the enrollment period is estimated to take approximately 10 months. Subjects will be followed for 90 days post implant or until resolution of any ongoing procedure-related adverse event(s). The total expected study duration is approximately 13 months.

There is no minimum requirement for enrollments per center for this study. Centers that enroll at a rate faster than others will be allowed to do so in order to maintain an adequate study enrollment rate. However, centers will be encouraged to enroll a maximum of 10 subjects to maintain a balance of patients among enrolling centers. This maximum center enrollment limit may be adjusted higher or lower during the course of the study in order to maintain balanced enrollment while facilitating completion of the trial within the estimated 10-month enrollment period.

2 Background and Justification

The study will be conducted using the Reveal DX and Reveal XT ICMs. Both devices are implantable, patient-activated and automatically-activated monitoring systems that record subcutaneous electrocardiogram (ECG). The ECG is a recording of the electrical activity of the heart and is useful in the diagnosis of cardiac conditions. Both Reveal ICM models store detailed information about the occurrence of cardiac arrhythmia episodes. Storage in the device is automatically triggered to collect data when the monitor detects a cardiac arrhythmia, such as ventricular tachycardia (VT), bradycardia, or asystole or can be triggered by the patient through the use of a Reveal Patient Assistant® device. The Reveal XT model has additional functionality allowing detection of Atrial Tachycardia and Atrial Fibrillation (AT/AF) episodes, viewing of long-term clinical trends

as well as heart rate histograms. The device(s) utilized during this study will be used according to current labeling.

The vast majority of current Reveal implant procedures in the U.S. are performed in a "facility" (hospital) setting on an outpatient basis.[1,2] The hospital setting is the traditional location for invasive cardiac device implants such as Implantable Pulse Generator (IPG), Implantable Cardioverter Defibrillator (ICD), and Cardiac Resynchronization Therapy (CRT) devices. The implant procedure for these devices has an inherent need for an advanced level of anesthesia and other surgical equipment due mainly to the required placement of transvenous cardiac leads which are inserted through the vascular system and are placed directly in the heart. Reveal implant procedures are minimally invasive and require only a small subcutaneous incision of approximately 2 cm in length; they do not require cardiac leads since the sensing electrodes are self-contained on the surface of the device. The implant procedure time for the Reveal device is significantly shorter than other cardiac devices, and many implants are currently being conducted with only local anesthesia.[3,4] These factors make the Reveal ICM an ideal device for successful implantation in the office or clinic setting.[5]

Implantation of ICMs began in the early 1990's.[6] Since that time the devices have reduced in size and grown in diagnostic capability. Most studies to date have focused on gathering evidence about the ICM's ability to diagnose cardiac arrhythmias as well as their cost-effectiveness. Throughout this research, the implant procedure itself has been documented to be safe with procedure-related infection rates reported between 0.5 and 4%, and other complications such as bleeding or pocket erosion reported at a similar rate. [3,4,7-22] The most important and relevant complications associated with ICM implantation are those which require surgical intervention to re-open or drain the device pocket. These events not only have the greatest impact on patient health and recovery, but in many cases require removal of the device. Surgical intervention may have a significant impact on the healthcare system costs associated with this procedure due to the increased incidence and length of hospital stays.[23]

The literature does not provide data regarding the timing of post-implant ICM complications; however, data from other implantable cardiac devices suggests the majority of pocket hematomas will occur within several days of the procedure and that 90 days is an established time frame for monitoring pocket infections.[24,25] Device infections have been reported in cardiac device studies beyond 90 days and up to several years after implant, however, this data has been reported for devices with intravascular leads and there is evidence to support that many of these infections are due to bacterial colonization of the device through the blood stream from infections that originated elsewhere in the body therefore not applicable to the ICM device.[26]

The intent of this study is to determine if in-office implant procedures have a comparable safety profile as well as similar resource and time requirements as procedures currently performed in the hospital setting. This information is intended to be used to help extend adequate reimbursement for the device implant procedure if it is performed in the non-facility site of service as well as to provide physicians with evidence to be able to assess any perceived or unknown risks or disadvantages of performing the implant procedure in a non-facility setting.

This study will characterize the implant-related adverse events and provide data on the time and resource needs for in-office / in-clinic implants to determine the feasibility of performing an ICM implant in this non-traditional implant setting.

All patients recruited to participate in the study will be indicated for the Reveal device and this procedure should be coverable under their specific health care benefit policy. This study is not intended to enroll patients who are not otherwise indicated for such a procedure and/or those whose reimbursement provider would not ordinarily cover and pay for such device implant.

3 System Description and Intended Use

The Reveal In-Office Implants study will use the Medtronic market released Reveal DX and Reveal XT ICM (or successor model). Subjects will receive either the Reveal DX or the Reveal XT as determined by the study investigator.

For a detailed description of the Reveal DX or Reveal XT system including information on software applications, instructions for use, storage and handling, and the recommended medical or surgical procedures involved, please refer to the appropriate Clinician Manual:

- Reveal DX 9528 Clinician Manual
- Reveal XT 9529 Clinician Manual

The Reveal DX and Reveal XT system components for this study are listed below, however, only the implant procedure of the Reveal ICM is being evaluated. Only the Reveal DX or the Reveal XT device and the CareLink® Programmer are required for this study. All other system components may be utilized at the discretion of the study investigator.

3.1 Reveal DX or Reveal XT Insertable Cardiac Monitor

The Reveal DX (model 9528) and the Reveal XT (model 9529) ICM are leadless devices that are typically implanted under the skin in the region of the thorax. Two electrodes on the body of the device continuously monitor the patient's subcutaneous ECG. The device memory can store up to 22.5 min of ECG recordings from the patient-activated episodes and up to 27 min of ECG recordings from automatically detected arrhythmia. When the ECG storage log is full, data from the most recent episode will overwrite data from the oldest stored episode of that type of arrhythmia.

A Vector Check tool is incorporated in the packaging of the Reveal ICM, which enables the implanting physician to select the most optimal implantation site while the device is still in the sterile package. Detailed information and implant instructions for the Reveal DX and XT devices can be found in their respective clinician manuals.

3.2 Medtronic Model 2090 CareLink® Programmer

The Medtronic Model 2090 CareLink® programmer with software model SW 0007 (or later releases) is used to set up and program the Reveal ICM.

It also allows the clinician to view, save, and print the device stored information including patient activated or automatically detected cardiac episodes.

3.3 Reveal Patient Assistant device

The Reveal Patient Assistant Model 9538 and Reveal XT Patient Assistant Model 9539 are handheld, battery-operated telemetry devices that enable the patient to record cardiac information in the Reveal ICM after experiencing symptoms of a possible cardiac event. When the patient experiences symptoms they may press a button on the device which will subsequently capture a pre-programmed duration of ECG data and store the episode for later viewing by the physician. The Reveal XT Patient Assistant Model 9539 Patient Assist also contains a query function which enables the patient to check the device and receive a notification when an arrhythmia has occurred or when the device status has changed. When the patient presses the query button on the Patient Assistant, the event indicator will light up if a status change or episode has occurred. The physician can select which device changes or cardiac episodes will provide the notification and can program the conditions that must be met before the notification is given.

3.4 Intended Use

The Reveal DX and Reveal XT Insertable Cardiac Monitors are implantable patient-activated and automatically-activated monitoring systems that record subcutaneous ECG and are indicated in the following cases:

- patients with clinical syndromes or situations at increased risk of cardiac arrhythmias
- patients who experience transient symptoms such as dizziness, palpitation, syncope and chest pain, that may suggest a cardiac arrhythmia

There are no known contraindications for the implant of the Reveal ICM. However, the patient's particular medical condition may dictate whether or not a subcutaneous, chronically implanted device can be tolerated. For full disclosure see the Reveal DX or Reveal XT Clinician Manual.

3.5 Regulatory Classification

This study will be conducted only in the United States. In the United States, the Reveal In-Office Implants study does not require an Investigational Device Exemption (IDE) as the Reveal ICM will be used per current labeling. Medtronic has determined that this study is a Non-Investigational Device study, which is exempt from 21 CFR Part 812. The clinical performance and safety of the market-released Medtronic Reveal ICMs are not being assessed in this post-market study.

The study will be conducted according to the Clinical Investigation Plan (CIP), the Declaration of Helsinki concerning medical research, ISO 14155 (part 1 and part 2) and in accordance to the laws and regulations of the countries in which it is conducted, and will take Good Clinical Practice (GCP) as guidance.

The principles of the Declaration of Helsinki have been implemented in this study by means of the patient informed consent process, IRB approval, study training, risk benefit

assessment, publication policy, etc. In addition, in accordance with the 2007 Food and Drug Administration Amendments Act (FDAA) and Declaration of Helsinki, this study will be publicly registered prior to first enrollment on http://clinicaltrials.gov/.

4 Methodology

The Reveal In-Office Implants study is a prospective, non-randomized, multi-center post-market clinical trial to evaluate the safety and feasibility of performing Reveal implants in an office or clinic setting. Subjects with established clinical indications for the Reveal ICM will be implanted using standard of care techniques and procedures. Implants will be required to take place in an office or clinic setting, rather than the traditional facility such as the hospital operating room, cardiac catheterization lab, or electrophysiology lab. Subject sedation will be limited to local anesthetics and/or oral anti-anxiety medications. The primary objective is to characterize the safety of in-office implants. Due to the low complication rate for this type of implant and practical limitations on the sample size, there are no formal hypotheses tested in this study. This study will characterize all procedure-related adverse events that occur within 90 days of the operative procedure. Data will be collected at implant and at a 30 and a 90-day post-implant visit as described in Section 5. Descriptive analyses of the results of this study will help establish whether the office or clinic is an appropriate implantation setting for the Reveal ICM.

4.1 Study Objectives

PRIMARY OBJECTIVE

Characterize the rate of procedure-related complications within 90 days of the implant procedure which require resolution by surgical intervention

SECONDARY OBJECTIVES

The following objectives are intended to provide additional data on the safety and feasibility of in-office implants:

- Characterize all procedure-related adverse events
- Characterize time and resource utilization for Reveal in-office implants
- Characterize techniques and procedures utilized during Reveal in-office implants
- Characterize physician satisfaction with Reveal in-office implants
- Characterize device functionality post-implant

4.2 Subject Selection

Inclusion Criteria:

1. Patient is indicated for continuous arrhythmia monitoring with an Implantable Cardiac Monitor
2. Patient is willing to undergo implant in clinic setting with only local anesthetic and/or oral anti-anxiety medications for sedation
3. Patient is 18 years of age or older

4. Patient is willing and able to provide consent and authorize the use and disclosure of health information
5. Patient is willing and able to comply with the protocol including the required follow-up

Exclusion Criteria:

1. Patient has reduced immune function or is otherwise at high risk for infection
2. Patient has had a recent (within 30 days) or otherwise unresolved infection
3. Patient is implanted or indicated for implant with a pacemaker, ICD, CRT, or hemodynamic monitoring system
4. Patient is participating in another clinical study that may have an impact on the study endpoints
5. Patient's life expectancy is less than 1 year
6. Patient is pregnant
7. Patient has unusual thoracic anatomy or scarring at the implant site which may adversely affect the success of the implant procedure

Patients will be screened to ensure they meet all of the inclusion and none of the exclusion criteria. Institutional Review Board (IRB) approval of the Reveal In-Office Implants study Clinical Investigation Plan and Patient Consent Form must be obtained prior to enrolling patients in the study.

4.3 Minimization of Bias

Potential sources of bias in this study may result from selection of subjects, treatment of subjects, and evaluation of study data. The following methods have been incorporated into the study to minimize potential bias.

- To minimize center bias, all sites will be encouraged to enroll 5 subjects and a maximum of 10 subjects to ensure a more balanced distribution of subject data.
- Centers will be selected that are geographically diverse
- Centers will utilize their own best practices for implant techniques and procedures so study results will reflect any current variations in treatment or implant technique.
- All centers will use the same version of the Clinical Investigation Plan and Case Report Forms (CRF).
- All study clinicians will be trained on and required to follow the Clinical Investigation Plan.
- All study clinicians and Medtronic personnel will be trained on their respective aspects of the study using standardized training materials.
- Subject history and demographics will be collected at baseline to identify possible differences that may affect the study endpoints.
- An independent qualified adjudication committee will be utilized to regularly review and adjudicate reported adverse events.
- All subjects with an attempted in-office Reveal implant will be followed until study exit, death, or Reveal device modification, whichever comes first.

5 Study Procedures

All clinical investigators managing the subject's health must be qualified practitioners and experienced in the diagnosis and treatment of subjects with cardiac arrhythmias. All implanting physicians must be experienced in the implantation of ICMs. Investigators and coordinators will be trained on their responsibilities, obtaining patient consent, and the Clinical Investigation Plan. Training on CRF completion will also be provided.

The following study equipment must be available at each center to support study activities:

- Medtronic Reveal DX or Reveal XT ICM
- Medtronic Model 2090 CareLink® Programmer
- Medical equipment and supplies necessary for surgical implant of a Reveal ICM
- Computer with high speed internet, Windows Internet Explorer for Remote Data Capture (RDC) system access, and appropriate storage media (3.5" floppy drive or USB) for accessing save-to-disk (S2D) data

The maintenance and calibration of the equipment used for this study will be assessed by the study center. Programmer calibration will not be monitored by the clinical investigation team, but will be maintained by Medtronic field representatives as per standard practice.

5.1 Informed Consent Process

Patient consent is defined as legally effective, documented confirmation of a subjects' (or their legally authorized representative or guardian) voluntary agreement to participate in a particular clinical investigation after information has been given to the subject on all aspects of the clinical investigation that are relevant to the subjects' decision to participate.

Each investigational center's Institutional Review Board (IRB) will be required to approve the Clinical Investigation Plan (CIP) and Patient Consent Form. It is the responsibility of the study sponsor to provide a Patient Consent Form that is in compliance with all applicable laws and regulations as local law and ISO 14155. Refer to Appendix E for the sample Patient Consent Form. Any changes to the Patient Consent Form must be approved first by Medtronic and then by the IRB reviewing the application before being used to consent a prospective study subject. The document(s) should be controlled (i.e. versioned and/or dated) to ensure it is clear which version(s) were approved by the IRB.

Prior to initiation of any study-specific procedures, subjects (or their legally authorized representative or guardian) must sign and date the HIPAA/data protection authorization/or other privacy language where required by law and the IRB and Medtronic approved Patient Consent Form. The Patient Consent Form and HIPAA/data protection authorization/or other privacy language where required by law, must be given to the subject (or their legally authorized representative or guardian) in a language he/she is able to read and understand.

The process of patient informed consent will not be conducted using coercion, or undue influence by the investigator or other center personnel. The process of obtaining patient informed consent shall:

- Avoid coercion and undue influence of subjects to participate
- Not waive or appear to waive subject's legal rights
- Use language that is non-technical and understandable to the subject
- Provide ample time for the subject to consider participation
- Include a dated signature of the subject acknowledging that their participation in the study is voluntary

In the event the subject cannot read and/or write, witnessed (impartial third party) informed consent will be allowed, provided detailed documentation of the process is recorded in the subject's case history and the witness signs and dates the Patient Consent Form. The original or a copy of the signed Patient Consent Form must be filed in the hospital/clinical chart or with the subjects' study documents. A copy of the signed Patient Consent Form and HIPAA/data protection authorization/or other privacy language where required by law must be provided to the subject. When a patient signs and dates the Reveal In-Office Implants study Patient Consent Form, he/she is considered a subject enrolled in the study. If the patient informed consent is obtained the same day the subject begins participating in study-related procedures, it should be documented in the subject's case history that consent was obtained prior to participation in any study-related procedures.

The Patient Consent Form and HIPAA/data protection authorization/or other privacy language where required by law must be available for monitoring and auditing. Any Medtronic Field personnel who support the procedures must be able to review the subject's signed and dated Patient Consent Form and verify its completeness prior to proceeding with the procedures. In the event the Medtronic Field personnel identify the Patient Consent Form as being incomplete, the procedure will not be allowed to occur until the informed consent of the subject can be adequately and appropriately obtained.

5.2 Data Collection

Table 1: Data Collection and Study Procedure Requirements at Subject Visits

STUDY PROCEDURE	Enrollment / Baseline[3]	Implant[4]	30-day Follow-up[5]	90-day Follow-up	Unscheduled[6]
Inclusion/Exclusion Criteria[1]	√				
Date of Consent and HIPAA/data protection Authorization[2]	√				
Demographics, Medical History Review	√				
Medications and Medication Changes	√	√	√	√	√
Height and Weight	√				
Implant Procedures/ Materials / Techniques		√			
Physician Survey		√			
Save to Disk / Collect R-Wave		√	√		as needed [7]
Technical Observations	as needed				
Adverse Events					
Exit Information					
System Modifications					
Study Deviations					
Death					
Equipment Utilization Survey	once per site				

1 Verify patient meets inclusion criteria and no exclusion criteria prior to consent
2 Patient Informed Consent must be collected prior to any study-related procedure
3 *Patient informed consent may be obtained from 0 - 30 days prior to implant*
4 Implant should occur from 0 – 30 days following the Baseline Visit
5 Follow-up visit should occur at least 30 days post implant – If subject exits the study prior to 30 days post-implant, all 30-day follow-up procedures should be completed at that time
6 Required if unscheduled visit occurs at study center after implant but prior to study exit. Includes wound check visit or other visit. Unscheduled visit can also be conducted by phone and may be used to collect exit information, if necessary
7 Save to Disk and R-Wave should only be recorded at this visit if the 30-day visit will not be conducted.

Clinical information will be collected by means of a web-based application tool, Remote Data Capture (RDC). This tool contains Electronic Case Report Forms (e-CRFs) which can be accessed via an Internet browser. Data inconsistencies will result in a discrepancy sent to the responsible center. The investigator is responsible for the preparation (review and signature) of the e-CRFs. Following database launch, a sample e-CRF will be available upon request. Paper worksheets of the e-CRFs will be available upon request.

Clinical data will be collected at:

- Baseline
- Implant
- 30-day Follow-up visit
- 90-day Follow-up visit
- All non-protocol required (unscheduled) cardiology follow-up visits (until subject is exited from the study)

A patient is considered enrolled if he/she meets the eligibility criteria and has signed the Patient Consent Form. Upon enrollment, the Baseline visit should be performed as soon as possible but must be performed within 30 days of the implant.

Furthermore, data will be collected at the occurrence of:

- System modifications (Reveal explanted / replaced / repositioned)
- Study deviations
- Adverse Events
- Technical observations
- Subject death
- Study Exit

5.3 Subject Follow-up Schedule

If the scheduled visit is not performed or performed outside the required visit window, this must be reported as a Study Deviation. See Table 2, below, for study visit windows.

Table 2: Study Visit Windows

Visit	Window *(days relative to implant*)*		
	Window Start (# of days)	Target (# of days)	Window End (# of days)
Enrollment	-30	-7	0
Baseline	-30	0	0
Implant*	0	0	0
30-day Follow-up	30	30	44
90-day Follow-up	90	90	104

**Implant is always day 0*

5.4 Enrollment

When a patient signs and dates the patient consent form, he/she is considered a subject enrolled in the study.

Subject enrollment must occur on the day of, or within 30 days prior to the implant procedure.

No study procedures may be performed prior to enrollment; **this includes the administration of procedure-related medications.**

5.5 Baseline

The date the subject signed the consent form and data protection authorization (if required) must be recorded at the Baseline visit. The Baseline assessment will be used to collect demographical data for each subject. In addition, detailed information including patient medical history, height, weight, and information about relevant medications (pain medication, anticoagulants, antiplatelet, antibiotics, anti-anxiety) will be collected.

The Baseline assessment must occur on the day of or within 30 days prior to the implant procedure.

5.6 Implant

The implant procedure will be performed according to standard implant technique. Implant guidelines can be found in the Reveal DX and Reveal XT Clinician Manuals and the study training material. There are no device programming requirements for subjects in this trial.

At implant, the device serial number will be recorded, and detailed information about the subject and the implant procedure will be collected:

- Timing of the procedure and administration of any procedure medication
- Resource and materials utilization
- Implant techniques/procedures
- Physician survey
- Device functionality

Any adverse events and any changes to the baseline medications will be reported.

Device functionality will be assessed by collection of the R-Wave amplitudes as recorded at the time of implant. In addition, a Full Final Interrogation of the device will be collected for each subject after final programming of the Reveal ICM prior to subject discharge by means of the 'Save-to-Disk' (S2D) feature.

5.7 30-Day Follow-up Visit

One scheduled Follow-up in-office visit will take place from 30-44 days post-implant. At this visit the subject and implant site will be examined for signs of adverse events. Any adverse events that are attributable to the implant procedure, as well as data on changes in medication use since implant will be recorded. To determine continued device functionality, the R-Wave amplitude and a Full Final Interrogation S2D will be collected for each subject.

5.8 90-Day Follow-up Visit

A final Follow-up visit will take place from 90-104 days post-implant. This visit may be conducted in the office or by telephone, if necessary. At this visit the subject and implant site will be examined for signs of adverse events. Any adverse events that are attributable to the implant procedure, as well as data on changes in medication use will be recorded. If this visit is conducted by phone, the subject will be asked to describe any signs and symptoms which may be indicative of an implant-related complication. If signs of a complication are suspected, the study site should request that the subject return to the clinic for an in-person visit. This visit should be recorded on the e-CRF as an Unscheduled Visit (see Section 5.9). Unless there is an unresolved adverse event, the subject should be exited from the study at the 90-day Follow-up visit.

5.9 Unscheduled Visit

After the implant procedure, any time the subject returns to the clinic for a visit other than those required by this protocol, an unscheduled visit e-CRF should be completed. At these visits, if examination of the implant site reveals any adverse events or if the subject reports any adverse events that are attributable to the implant procedure, this data should be recorded on an adverse event e-CRF. In addition, any changes in medication use by the subject since implant should also be recorded.

If there is a need to exit the subject prior to the 30-day visit, the unscheduled visit e-CRF should be completed as well as the exit e-CRF. In this circumstance continued device functionality should be verified by collection of a Full Final Interrogation S2D of the device and recording of the R-Wave amplitude on the unscheduled visit e-CRF. Interrogation of the device and recording of the R-Wave amplitude is only required once after the day of implant (day 1 to day 104). If this information has been or will be collected at the 30-day visit, it should not be collected at the time of the unscheduled visit.

5.10 Adverse Event

With the exception of unavoidable adverse events (AEs) defined in Table 3, all AEs related to the procedure must be reported to Medtronic. Adverse Events must be reported according to the instructions in Table 7 of Section 8.6.

Each AE must be reported separately and will include a description of the event, the diagnosis, the date of event onset, the relatedness of the event, diagnostic tests and procedures performed, actions taken as a result of the event, and/or the outcome of the event. Any medication associated with the treatment of an adverse event must also be reported.

For any changes in status of a previously reported AE (i.e. change in action, change in outcome, change in relatedness), an Adverse Event Update must be reported. All AEs must be followed until the adverse event has been resolved, is ongoing with no further actions to be taken, the subject exits the study or until study closure, whichever occurs first.

For a listing of procedure related AEs, please refer to Section 8, Table 3 and Section 9.

5.11 System Modification

Whenever the device is explanted, replaced or repositioned during the follow-up period; this is considered a system modification**.** Any modification to the implanted system must be reported on the e-CRFs.

If the system modification was caused by a procedure-related AE, an AE should be reported on the AE e-CRF.

In the event of a system modification the subject should be exited from the study.

5.12 Technical Observation

A technical observation is defined as a failure, malfunction or function not according to design intent. Technical observations should be documented on the e-CRF. Procedure related events that adversely affect the subject should be reported as an AE and not a Technical Observation.

5.13 Study Exit

Upon completion of the trial, study exit and reason for subject withdrawal must be documented on the e-CRF.

Study exit will typically take place on the day of and immediately following the 90-day follow-up visit, however could be completed at any point in the trial if unusual circumstances occur. If study exit occurs at any time other than the 90-day follow-up visit, record this information on an unscheduled visit e-CRF.

Other reasons subjects may be exited from the trial include, but are not limited to the following:

- Subject requested withdrawal from the study
- Inclusion/exclusion criteria not met prior to implant attempt but Patient Consent Form was signed
- Investigator withdrew subject from the study
- No implant attempted within 30 days post enrollment
- Implant attempted, however device was not implanted – no incision was made
- System modification
- Subject is Lost to Follow-up
- Subject receives an ICD, pacemaker or CRT-device

If an implant is attempted (incision was made) but no device is implanted, the subject should remain in the study until the 90-day follow-up visit or another reason for study exit is met.

If the subject has ongoing procedure-related adverse events the subject should continue in the trial and complete the exit procedures after that event has resolved or after the investigator determines that the event is ongoing and no further actions will be taken.

Subject Follow-up after Withdrawal
Upon withdrawal from the study, no further study data will be collected or study visits will occur for the subject. The subject will continue to receive standard medical care. It will be the physician's and/or subject's decision to continue use of the Reveal system. The Reveal device will not be, explanted, replaced or re-positioned as a part of this study and any further follow-up of the subject and the device will be at the physician's and/or subject's discretion.

Subject lost to follow-up
In the case that the subject is determined to be lost to follow-up, details of a minimum of two attempts and the method of attempt (e.g., one letter and one phone record or two letters) to contact the subject must be documented. In addition, the investigator should follow the regulations set forth by the governing IRB.

Subject Death
Data collection for subject death is discussed in Section 8.3.

5.14 Medications

The study will collect all medications that may impact the implant procedure as well as those medications that may be used to treat potential procedure-related adverse events.

- All oral anti-coagulants and antiplatelets (Aspirin/ASA, Clopidogrel, Dipyridamole)

- All anti-anxiety medications
- All antibiotics
- All pain medications or analgesics
- All procedure related anesthesia
- Any other procedure-related medications, include topical medication, but exclude antiseptic agents used to clean the surgery site (these agents will be collected on the implant form)

For all medications listed above, the start date and stop date will be collected.

For all medications used for the purpose of the implant procedure, the time of administration relative to implant and route of administration, in addition to the start and stop date, will be collected

Titration of all anticoagulant medications will be documented.

There are no medications that are required for this study. Only Investigational medications are excluded from use during this study.

5.15 Equipment Utilization Survey

Equipment used for the purpose of the implant procedure will be documented on one e-CRF per study site. This survey will also contain questions regarding the structure of the procedure room. This e-CRF may be revised and updated by the investigator throughout the course of the trial to create a final, 'best-practice' list at the end of the study for that site.

6 Device Storage, Handling and Traceability

The Reveal devices used in this study are commercially available. It is not a requirement to return explanted devices to study management. If a subject dies during the study, it is recommended to explant the Reveal device whenever possible and return to Medtronic for analysis. Please follow applicable local law procedures. Prior to explant, the system shall be interrogated and a Full Final Interrogation performed (S2D) when possible. If any system component is returned to Medtronic, internal return product reporting systems may be used to gather additional information about the returned device/component.

7 Study Deviations

A study deviation is defined as an event within a study that did not occur according to the Clinical Investigation Plan or the Clinical Trial Agreement (CTA). Prior approval by the Medtronic Study Leader is expected in situations where the investigator anticipates, contemplates, or makes a conscious decision to deviate. Prior approval is not required when a deviation is necessary to protect the life or physical well being of a subject in an emergency or in unforeseen situations beyond the investigator's control (*e.g.* subject

failure to attend scheduled follow-up visits, inadvertent loss of data due to computer malfunction, inability to perform required procedures due to subject illness).

All deviations from the Clinical Investigation Plan shall be documented and explained, regardless the reason for the deviation. The documentation must include the justification for the deviation. Study deviations mentioned below will be captured during the course of the study. Planned deviations in the study shall be submitted for approval to the sponsor and all deviations must be reported through completion of a Study Deviation e-CRF, including, but not limited to:

- Patient consent deviations
- Subject did not meet inclusion or exclusion criteria
- Visit compliance deviations (e.g. missed visit or visit outside window)
- Regulatory compliance deviations
- Source document deviations
- Protocol required training deviations
- Adverse event reporting deviations

Study deviations must be reported to Medtronic, regardless of whether medically justifiable, pre-approved by Medtronic, or taken to protect the subject in an emergency.

Reporting of study deviations must comply with IRB policies and/or local laws or regulatory agency requirements and must be reported to Medtronic as soon as possible upon the center becoming aware of the deviation. Refer to Investigator Reports, Section 15.2 for timeframes for reporting to Medtronic and/or regulatory bodies. Medtronic is responsible for analyzing deviations, assessing their significance, and identifying any additional corrective and/or preventive actions (e.g. amend the Clinical Investigation Plan, conduct additional training, terminate the investigation, etc.). Repetitive or serious investigator compliance issues may initiate a corrective action plan with the investigator and site, and in some cases, necessitate suspending enrollment until the problem is resolved or potentially terminating the investigator's participation in the study. Medtronic will provide center-specific reports to investigators summarizing information on deviations that occurred at the investigational site on a periodic basis.

8 Adverse Events

Timely, accurate, and complete reporting and analysis of safety information for clinical studies are crucial for the protection of subjects, investigators, and the sponsor. Reporting and analysis of safety data are mandated by regulatory authorities worldwide. Medtronic has established Standard Operating Procedures (SOPs) in conformity with worldwide regulatory requirements to ensure appropriate reporting of safety information. This study is conducted in accordance with these SOPs and regulations. Since the safety reporting requirements and classification systems vary for each regulatory agency, requirements from all geographies are taken into account for the collection and reporting of safety information. There are no anticipated adverse event (AE) stopping rules planned for this study.

8.1 Adverse Event Collection Requirements and Definitions

Adverse Event Collection Requirements

All procedure-related AEs will be collected throughout the study duration. AE collection will begin when the subject is enrolled in the study and undergoes a Reveal implant attempt. The AE will be reported to Medtronic on an Adverse Event Form. Documented pre-existing conditions are not considered AEs unless the nature or severity of the condition has worsened.

Unavoidable Adverse Events, listed in Table 3, below, need not be reported.

Each AE must be reported separately and will include a description of the event, the diagnosis, the date of event onset, the relatedness of the event, diagnostic tests and procedures performed, actions taken as a result of the event, and/or the outcome of the event. Any medication associated with the treatment of an adverse event must also be reported.

Subject deaths are required to be reported. Refer to Section 8.3 for Subject Death collection and reporting requirements.

<u>Processing updates and resolving Adverse Events</u>

For any changes in status of a previously reported AE (i.e. change in action, change in outcome, change in relatedness), an Adverse Event Update must be reported. All AEs must be followed until the adverse event has been resolved, is ongoing with no further actions to be taken, the subject exits the study or until study closure, whichever occurs first.

Table 3: Adverse Event Definitions

Definition	Description
**General**	
**Adverse Event** (ISO 14155-1)	Any untoward medical occurrence in a subject<br>NOTE: This definition does not imply that there is a relationship between the adverse event and the device under investigation.
**Seriousness**	
**Serious Adverse Event (SAE)** (ISO 14155-1)	An Adverse Event that<br>a) led to a death,<br>b) led to a serious deterioration in the health of the subject that<br>• Resulted in a life-threatening illness or injury<br>• Resulted in permanent impairment of a body structure or a body function<br>• Required in-patient hospitalization or prolongation of existing hospitalization<br>• Resulted in medical or surgical intervention to prevent permanent impairment to body structure or a body function<br>c) led to fetal distress, fetal death or a congenital abnormality or birth defect.

<table>
<tr><td>Complication</td><td>An adverse event that results in death, involves any termination of significant device function, or requires invasive intervention.</td></tr>
<tr><td>Observation</td><td>Any adverse event that is not a complication</td></tr>
<tr><td colspan="2">Relatedness</td></tr>
<tr><td>Procedure Related</td><td>An adverse event that occurs due to any procedure related to the implantation or surgical modification of the device</td></tr>
<tr><td colspan="2">Timing</td></tr>
<tr><td>During Implant AE</td><td>An adverse event that occurs during implant, after skin incision</td></tr>
<tr><td>Post-Implant AE</td><td>An adverse event that occurs after the completion of skin closure for the implant</td></tr>
<tr><td>Unavoidable AE</td><td>An Adverse Event inherent to a surgical procedure that is expected to occur in all subjects for a projected duration according to the Investigator’s opinion, including, but not limited to:
<table>
<tr><th>Event Description</th><th>Time Frame (Hours) from the Surgical Procedure</th></tr>
<tr><td>Anesthesia related nausea / vomiting</td><td>24</td></tr>
<tr><td>Low-grade fever (<100°F or <37.8°C)</td><td>48</td></tr>
<tr><td>Pocket site / Incisional pain</td><td>72</td></tr>
<tr><td>Mild to moderate bruising / ecchymosis</td><td>168</td></tr>
<tr><td>Sleep problems (insomnia)</td><td>72</td></tr>
<tr><td>Back pain related to laying on table</td><td>72</td></tr>
<tr><td>Shoulder pain/discomfort/stiffness related to shoulder immobilization during procedure</td><td>72</td></tr>
<tr><td></td><td></td></tr>
</table></td></tr>
<tr><td colspan="2">Other</td></tr>
<tr><td>Implant Site Infection</td><td>An implant site infection must meet the following criteria:
Subject has at least one of following:
o Purulent drainage from the incision
o Organisms isolated from an aseptically obtained culture of fluid or tissue from the incision
o At least one of the following signs or symptoms of infection: pain or tenderness, localized swelling, redness, or heat, and incision is deliberately opened by surgeon and is culture positive or not cultured. A culture-negative finding does not meet this criterion.
o Diagnosis of implant site infection by the surgeon or attending physician based on clinical evidence including: erythema, warmth, fluctuance, wound dehiscence, erosion, or tenderness at the implant site.[26]</td></tr>
</table>

8.2 Adverse Event Classification and Reporting

Adverse Events will be classified according to the definitions provided in the previous section. By completing the AE electronic Case Report Form, geography required regulatory definitions and classifications will be satisfied. Upon receipt of AEs at Medtronic, a Medtronic representative will review the AE for completeness and accuracy and when necessary will request clarification and/or additional information from the Investigator. Medtronic will utilize MedDRA, the Medical Dictionary for Regulatory Activities, to assign a MedDRA term for each AE based on the information provided by the investigator. Regulatory and IRB reporting of AEs will be completed according to local regulatory requirements. Refer to Table 7 for a list of Investigator reporting requirements and timeframes. It is the responsibility of the Investigator to abide by the AE reporting requirements stipulated by the IRB.

For emergency contact regarding a SAE, contact the Clinical Research Specialist immediately (refer to the study contact list provided in the center's study documents binder/investigator site file or refer to the contact information provided on the title page). Adverse Events and Deaths will be classified according to the standard definitions as outlined in Table 4 below:

Table 4: Adverse Event Responsibilities

What is Classified	Who Classifies	Classification Parameters
Timing of the Event[1]	Investigator	Implant or Post-implant
Relatedness[2]	Investigator	Study Procedure related
Seriousness	Investigator	Serious Adverse Event or not Serious Adverse Event
	Sponsor	Complication or Observation
Diagnosis/key term[1]	Investigator	Based on presenting signs and symptoms and other supporting data
	Sponsor	MedDRA term assigned based on the data provided by Investigator
Adjudicates All of the Above[1]	Adverse Event Advisory Committee (AEAC)	

[1] The AEAC may not adjudicate the timing or the key term of the event
[2] The AEAC will also classify the relatedness of each procedure-related AE to an implant site infection

8.3 Death Classifications and Reporting Requirements

All subject deaths must be reported by the investigator to Medtronic on a Subject Death form as soon as possible after the investigator first learns of the death.

In the event of a subject death, and where required, it is requested that the implanted system component be explanted as soon as possible and returned to Medtronic for analysis. Please follow applicable local law procedures.

Prior to explant, the system component shall be interrogated and a Full Final Interrogation performed (S2D) when possible. If the system component is not interrogated, an explanation must be entered on the Subject Death form. If the system component is returned to Medtronic, internal return product reporting systems may be used to gather additional information about the returned device/component.

A copy of the death certificate, if available and allowed by state/local law, should be sent to the in-house Medtronic clinical study team. When a death occurs in a medical institution, a copy of the physician's dictated death summary report and all relevant hospital records should be sent to the in-house Medtronic clinical study team, if available. If an autopsy is conducted, the autopsy report should also be sent to the in-house Medtronic clinical study team. When the death occurs at a remote site, it is the investigative center's responsibility to attempt retrieval of information about the death. Additionally, device disposition information should be updated. In summary, the following data will be collected:

- Date of death
- Detailed description of death
- Cause of death

- Device Interrogation Save-to-Disk (if available)
- Relatedness to system
- Device disposition information
- Death certificate (if available and/or allowed by state/local law)
- Death summary/hospital records (if available and allowed by state/local law)
- Autopsy report (if available and allowed by state/local law)

Sufficient information will be required in order to properly classify the subject's death. The Investigator shall classify each subject death per the following definitions:

Table 5: Death Classification Definitions

Classification	Definition
Cardiac Death	A death directly related to the electrical or mechanical dysfunction of the heart.
Non-cardiac Death	A death not classified as a cardiac death.
Sudden Cardiac Death (SCD)	Natural death due to cardiac causes, heralded by abrupt loss of consciousness within one hour of the onset of acute symptoms; preexisting heart disease may have been known to be present, but the time and mode of death are unexpected. If time of onset cannot be determined, SCD will alternatively be defined as any unexpected cardiac death occurring out of the hospital or in the emergency room as dead on arrival.
Non-sudden Cardiac Death	All cardiac deaths that are not classified as sudden deaths, including all cardiac deaths of hospitalized subjects on inotropic support.
Unknown	Unknown death classification is intended for use only when there is insufficient or inadequate information to classify the death.

Table 6: Subject Death Classification Responsibilities

What is Classified	Who Classifies	Classification Parameters
Relatedness	Investigator	• Device related, Reveal XT or Reveal DX<br>• Study Procedure related
Death Classification	Investigator	Sudden Cardiac, Non-sudden Cardiac, Non-cardiac, Unknown
Adjudicates All of the Above	Adverse Event Advisory Committee (AEAC)	

Regulatory and IRB reporting of Subject Deaths will be completed according to local regulatory requirements. Refer to Table 7, below, for a list of investigator reporting requirements and timeframes.

8.4 Adverse Event Advisory Committee Review (AEAC)

At regular intervals, an independent Adverse Event Advisory Committee (AEAC) will conduct a medical review of adverse events and deaths for subjects participating in the study. The AEAC will consist of a minimum of three (3) non-Medtronic employed physicians, including an AEAC chairperson. At least three AEAC members must adjudicate, at a minimum, all deaths and serious AEs related to the study procedure.

Medtronic personnel may facilitate and participate in an AEAC meeting but will be non-voting members.

Medtronic will provide the AEAC with the Investigator's description and classification of each AE in addition to the MedDRA key term. The AEAC is responsible for reviewing the investigator's assessment and classification of each event, and adjudicating a final classification.

If the AEAC disagrees with the investigator's classification of the event, the rationale will be provided to the investigator. If the investigator agrees with the AEAC's adjudication, the case report form documenting the AE or AE update will be updated accordingly.

If the investigator does not agree with the AEAC's adjudication classification, both determinations will be provided within the final report. However, the AEAC's adjudication will be used for data analysis.

8.5 Medical Device Reporting (MDR) Requirements for User Facilities

Per FDA regulations, Device User Facilities are required to report Medical Device Reports (MDR) on market approved products (21 CFR 803, subpart C) A Device User Facility is defined as a hospital, an ambulatory surgical facility, a nursing home, an outpatient treatment facility, or an outpatient diagnostic facility which is not a physician's office.

8.6 Investigator Adverse Event Records and Reporting Requirements

Adverse Events will be recorded and reported according to local regulatory requirements. Refer to Table 7, below, for investigator adverse event reporting requirements. It is the responsibility of the Investigator to abide by the adverse event reporting requirements stipulated by the centers' IRB.

Table 7: Investigator Adverse Event Reports

General Requirements:		
**Report**	**Submit To**	**Description/Constraints**
Death	Sponsor	Submit as soon as possible after the investigator first learns of the event
	IRB	Submit to IRB per local reporting requirements
Serious Adverse Events (SAE)	Sponsor	Submit on e-CRF as per protocol requirements and as required per local reporting requirements
	IRB	Submit to IRB per local reporting requirements
Procedure Related Adverse Events	Sponsor	Submit on e-CRF as per protocol requirements and as required per local reporting requirements
	IRB	Submit to IRB per local reporting requirements

9 Risk Analysis

Medtronic follows rigorous Quality Assurance and Control procedures throughout the life of a product, from the business analysis phase through development, market release, and post-market surveillance. The risk analysis process for the Reveal In-Office Implants study is being performed in accordance with ISO 14971, and will ensure that the level of risk is acceptable prior to starting the clinical study.

All systems and implantable devices in this study are market-released and used according to medical, technical and ethical standards without any change to the intended use of the device or any contraindications to current FDA labeling. Devices should be handled according to the Clinician Manual.

This study is designed to evaluate the clinic or office as an appropriate setting for the Reveal ICM implant. There is no evidence of increased risk of infection or complication due to in-office implants. This study will help to evaluate those objectives.

There are potential risks and discomforts associated with receiving a subcutaneous cardiac monitor. Standard risks as described in the Reveal ICM Clinician Manuals:

- Device rejection phenomena including local tissue reaction
- Device migration
- Pocket infection
- Erosion through the skin

Other risks
If the subject is female and is or becomes pregnant there may be unforeseen risk to the subject or to the embryo or fetus.

9.1 Risk Minimization

The risks identified above will be minimized by careful assessment of each subject prior to, during, and after implant of the Reveal ICM. The study will also require physicians to utilize only local anesthesia and/or oral anti-anxiety medications and only those sites with extensive Reveal implant experience will be selected to participate. After implantation, subjects in the Reveal In-Office Implants clinical study will be followed for at least 90 days to evaluate any potential implant-related adverse events. Additionally, at any time during the procedure or the study, the physician is allowed to deviate from the protocol, with justification, if there are any subject safety concerns. This must be reported as a Study Deviation.

9.2 Potential Benefits

The Reveal In-Office Implants study may offer no benefit. The potential benefits of having the Reveal ICM implanted in the office setting include a reduction in the time required for implant scheduling, preparation, procedure, and post-procedure recovery. The information gained from this study could result in the improved procedures for the implant of ICM's allowing more patients to successfully benefit from earlier detection of cardiac arrhythmia's resulting in more optimal medical treatment.

10 Planned Study Closure, Early Termination of Study or Suspension

10.1 Planned Study Closure

Study Closure is a process initiated by distribution of an initial study closure letter. Study closure is defined as closure of a clinical study that occurs when Medtronic and/or regulatory requirements have been satisfied per the Clinical Investigation Plan and/or by a decision by Medtronic or regulatory authority, whichever occurs first. The study closure process is complete upon distribution of the Final Report or after final payments, whichever occurs last. IRB re-approvals are required until the overall study closure process is complete.

10.2 Early Termination or Suspension

Early Termination of the Study is the closure of a clinical study that occurs prior to meeting defined endpoints. This is possible for the whole study or a single center.

Study Suspension is a temporary postponement of study activities related to enrollment and distribution of the product. This is possible for the whole study or a single center.

Criteria

Study Termination or Suspension

Possible reasons for considering study suspension or premature termination of the study may include:

- Adverse events associated with the system or procedure under investigation which might endanger the safety or welfare of the subject
- Observed/suspected performance different from the product's design intent
- Decision by Medtronic
- Technical issues during the manufacturing process

Investigator/Center Termination or Suspension

Possible reasons for clinical investigator or center termination or suspension include but are not limited to:

- Failure to obtain initial IRB/Head of Medical Institution approval or annual renewal of the study
- Consistent non-compliance to the clinical investigation (e.g. failure to adhere to inclusion/exclusion criteria, failure to follow subjects per scheduled follow-ups, etc.)
- Lack of enrollment
- Noncompliance to regulations and the terms of the Clinical Trial Agreement (e.g. failure to submit data in a timely manner, failure to follow-up on data queries and monitoring findings in a timely manner, etc.)
- IRB suspension of the center
- Fraud or fraudulent misconduct is discovered (as defined by local law and regulations)
- Investigator request (e.g. no longer able to support the study)

Procedures

If Medtronic terminates or prematurely suspends the study:

Medtronic will promptly inform the clinical investigators of the termination or suspension and the reasons and inform the regulatory authorities (where required per regulatory requirements).

In the case of study termination or suspension for reasons other than a temporary IRB approval lapse, the investigator will promptly inform the IRB.

- In the case of study termination, the investigator must inform the subjects and may inform the personal physician of the subjects to ensure appropriate care and follow-up is provided.
- In the case of a study suspension, subjects already enrolled should continue to be followed out of consideration of their safety, rights and welfare.

If the investigator terminates or suspends the study without prior agreement of Medtronic:

- The investigator will promptly inform Medtronic and provide a detailed written explanation of the termination or suspension.
- The investigator will promptly inform the institution (where required per regulatory requirements).
- The investigator will promptly inform the IRB.

If the IRB terminates or suspends its approval of the study:

- The investigator will promptly inform Medtronic and provide a detailed written explanation of the termination or suspension within 5 business days.
- Subject enrollment must stop until the IRB suspension is lifted.
- Subjects already enrolled should continue to be followed in accordance with IRB policy or its determination that an overriding safety concern or ethical issue is involved.
- The investigator will inform his/her institution (where required per local requirements).
- The investigator will promptly inform the subjects and/or the personal physician of the subjects, with the rationale for the study termination or suspension.

11 Statistical Methods and Data Analysis

11.1 Primary Objectives

Primary Objective

Characterize the rate of procedure-related complications within 90 days of implant which require resolution by surgical intervention

For this study there is no formal hypothesis or performance criterion tested for this objective. Instead, the rate of these complications will be characterized by computing a point estimate and one-sided upper 95% confidence interval for the rate of procedure-related complications requiring surgical intervention 90 days post-implant.

For purposes of the analyses described below, “events” are defined as procedure-related complications requiring surgical resolution or procedure-related complications resulting in SAEs.

Sample Size and Rationale

The table below shows the expected 90-day event rate and associated upper 1-sided 95% confidence interval for the 90-day rate of procedure-related complications requiring surgical intervention for several study scenarios when 65 subjects are implanted. Each scenario is based on 10,000 simulations of the study based on the following assumptions:

- 65 subjects will be enrolled and undergo an in-office Reveal implant
- 17% of subjects enrolled in the study will be diagnosed and have the Reveal explanted by the 90-day follow-up visit (Based on a study a study of 392 Reveal patients studied by Brignole et. al (2006),[27] 17% had diagnoses within 3-months)

- Two percent of subjects will exit the study for other reasons during the 90-day follow-up period
- Reveal explant and study exit rates are assumed constant across the 90-day follow-up period and are independent
- Assumed 90-day rate of procedure related complications requiring surgical intervention rates of 1.1%, 3.5%, 4.5% and 6.5% with the majority of events occurring within the first 30 days following implant
- Kaplan-Meier method used to estimate event rate and 1-sided upper 95% confidence interval for event rate at 90 days post-implant if at least 1 event is observed
- Exact binomial upper 95% confidence interval for the event rate if zero events are observed during the 90-day follow-up period

Table 8: 90-Day Event Rates

90-Day Event Rates Based on 10,000 Simulations of the Reveal In-Office Study						
	Assumed Event Rate (%)					
**Scenario**	**30-day**	**90-day**	**Expected 90-day Event Rate (%)**	**Expected upper 1-sided 95% CI (%)**	**P(CI < 10%)**	**P(CI < 15%)**
1	0.9[1]	1.1[1]	1.11	7.18	0.921	0.999
2	3.0	3.5	3.51	10.3	0.528	0.931
3	4.0	4.5[2]	4.47	11.6	0.388	0.853
4	6.0	6.5	6.52	14.1	0.174	0.612

[1]Based on complication rate from infections related to ICD implant reported by Al-Khatib et. al. 2005[25]
[2] Based on 4 procedure-related complications in 85 reveal subjects reported by Krahn et. al. 1999[1]

Expected event rates and associated 1-sided 95% upper confidence are shown in the table above based on 10,000 simulations of the Reveal In-Office Implants study for each of four study scenarios. For example, scenario one shows that if the true 30-day and 90-day event rates are 0.9% and 1.1% respectively, the expected 90-day event rate based on simulation is 1.11% with an expected upper 1-sided 95% confidence interval of 7.18%. In this scenario, there is a 92.1% and a 99.9% chance that the upper 1-sided confidence interval would by less than 10% and 15% respectively.

Experimental Design
Procedure-related adverse events are identified and reported on Adverse Events e-CRFs as they occur. Investigators will also record whether the adverse event was a SAE and record all interventions made to resolve the adverse event on Adverse Event or Adverse Event Update e-CRFs. For each adverse event reported, the AEAC will classify the seriousness of the adverse event, relatedness of the adverse event to the procedure, relatedness of the adverse event to infection, and determine if each adverse event is a complication or observation.

Analytical Methods

For purposes of this analysis, events are defined as procedure-related adverse events that result in surgical intervention or procedure-related events that result in an SAE as determined by the AEAC. The primary analysis will use the Kaplan-Meier method to estimate the event rate and associated 95% upper 1-sided confidence interval at 90 days post implant. In addition, a Kaplan-Meier plot of the event rate and upper 1-sided confidence interval will be constructed from the day of implant through day 90. For this analysis, days of follow-up will be computed as the days from implant to the onset date of the first event for subjects with events. For subjects without an event, days of follow-up will be calculated as days since implant to last follow-up date. The last follow-up date will be defined as the date of the 90-day visit, death, exit, or data cutoff date (if used), whichever comes first. Days of follow-up for subjects who initially have an unsuccessful in-office implant(s), but later have a successful implant will be calculated from the date of the successful implant to the date of last follow-up or event date. However, if an event occurs during an unsuccessful in-office implant procedure, days of follow-up will be set to zero for the analysis. All subjects with an attempted in-office Reveal implant will be included in this analysis.

If zero procedure-related complications resulting in surgical intervention or SAE are observed during the study, the 1-sided 95% upper confidence interval for the 90-day event rate will be calculated using an exact binomial confidence. For this calculation, the denominator will be the number of subjects with at least 90 days of follow-up.

11.2 Secondary Objectives

The secondary objectives will be evaluated to gain additional information about the feasibility of implanting the Reveal device in the office setting. There are no performance criteria or pre-specified hypotheses for these secondary objectives.

Secondary Objective #1

Characterize all procedure-related adverse events

Analytical Methods

All adverse events considered procedure-related by the investigator will be collected on e-CRFs as they occur. The AEAC will adjudicate each event and classify it as procedure-related, infection-related, serious or not serious, and whether the event was a complication or observation. In addition, interventions made to resolve each adverse event will also be reported on the Adverse Event e-CRFs. The total number of procedure-related complications and observations will be summarized. Additionally, the number of these events requiring surgical interventions or other therapy (e.g. antibiotic therapy) and the relatedness of the events to infection will be reported. All adverse events will also be summarized by AE key term and all adverse events including both investigator and AEAC classification will be listed.

Secondary Objective #2

Characterize time and resource needs for in-office Reveal implants

Analytical Methods
Procedure duration as well as the types of personnel and resources needed for in-office Reveal implant will be collected on the implant case report form. This information will be summarized using summary statistics and/or graphical methods.

Secondary Objective #3

Characterize implant techniques and procedures utilized during in-office Reveal implants

Analytical Methods
Techniques and procedures utilized during in-office Reveal implants will be collected on case report forms. This information will be summarized using summary statistics and/or graphical methods.

Secondary Objective #4

Characterize physician satisfaction with Reveal in-office implants

Analytical Methods
After each implant, the implanting physician will complete a physician satisfaction questionnaire. Summary statistics and/or graphical methods will be used to summarize all questionnaire responses.

Secondary Objective #5

Characterize device functionality post-implant

Analytical Methods
Device functionality post-implant will be characterized by collecting R-wave amplitudes on the day of implant and at the 30-day follow-up visit. Following each implant, R-wave amplitude will be collected and recorded on the Implant e-CRF. In addition, R-wave amplitude will be collected and recorded at the 30-day follow-up visit or at an unscheduled visit should the subject exit the study prior to the 30-day follow-up visit. Summary statistics and/or graphical methods will be used to summarize the R-wave amplitude.

Interim Report
An interim analysis of the primary and key secondary objectives may be conducted and utilized in conjunction with submission to reimbursement agencies in support of seeking appropriate reimbursement for Reveal implants done in the office or clinic setting. A data cut-off date for this interim analysis will be documented at the time of the interim analysis. There will be no adjustment to the confidence interval coverage probability for this interim analysis in this study.

12 Data and Quality Management
Data will be collected using an electronic data management system for clinical trials. CRF data will be stored in a secure, password-protected database which will be backed up nightly. Data will be reviewed using programmed and manual data checks. Data queries will be made available to centers for resolution. Study management reports may

be generated to monitor data quality and study progress. At the end of the study, the data will be frozen and will be retained indefinitely by Medtronic.

Procedures in the Clinical Investigational Plan require source documentation. In some cases, items on the CRFs may be considered source as long as there is evidence of the visit in the subject's record. Even when the CRF may be considered as source, an alternate method of source documentation is always strongly encouraged.

Save to disk data collected at office visits will be sent to Medtronic. Upon receipt, device data will be maintained with databases and retrieved for analysis and reporting.

The sponsor or a regulatory authority may audit the study center to evaluate the conduct of the study. The clinical investigator(s)/institution(s) shall allow trial related monitoring, audits, Ethics Board review and regulatory inspection(s) by providing direct access to source data/documents.

13 Warranty Information

Warranty information is provided in the product packaging for the Reveal ICM and Patient Assist Devices and additional copies are available upon request.

14 Monitoring

It is the responsibility of Medtronic to ensure proper monitoring of the study per regulations. Appropriately trained Medtronic personnel or delegates appointed by Medtronic may perform study monitoring at the study center in order to ensure that the study is conducted in accordance with the CIP, CTA, and applicable regulatory requirements. Medtronic must therefore be allowed access to the subjects' clinic and hospital records when so requested as per the Subject Informed Consent, Privacy Authorization and CTA.

14.1 Monitoring Visits

Any site monitoring will be conducted according to a separate monitoring guidance for this study.

15 Required Records and Reports

15.1 Investigator Records

The investigator is responsible for the preparation and retention of the records cited below. All of the below records, with the exception of case history records and case report forms, should be kept in the Investigator Site File (i.e., the study binder provided to the investigator) or Subject Study Binder. CRFs may be maintained and signed electronically within the electronic data capture system during the trial. The following records are subject to inspection and must be retained for a period of two years (or

longer as local law or hospital administration requires) after the date on which the investigation is terminated.

- All correspondence between the IRB, sponsor, monitor
- Subject's case history records, including:
  - Signed and dated informed consent form
  - Observations of adverse events/adverse device effects
  - Medical history
  - Implant and follow-up data
  - Documentation of the dates and rationale for any deviation from the protocol
- Signed and dated CRFs
- All approved versions of the Investigation Plan
- Signed and dated Clinical Trial Agreement
- Investigators current curriculum *vitae*
- Delegated task list
- IRB approval documentation. Written information that the investigator or other study staff, when member of the IRB did not participate in the approval process
- Study training records for site staff
- Any other records that FDA and local regulatory agencies require to be maintained
- Final Study Report including the statistical analysis

15.2 Investigator Reports

The investigator is responsible for the preparation (review and signature) and submission to the sponsor of all case report forms, adverse events, deaths, and any deviations from the investigation plan. If any action is taken by an IRB with respect to this clinical study, copies of all pertinent documentation must be forwarded to Medtronic in a timely manner. Reports are subject to inspection and to the retention requirements as described above for investigator records.
Safety data investigator reporting requirements are listed in Section 8.6 of the Adverse Event section.

The investigator shall prepare and submit in a complete, accurate and timely manner the reports listed in this section.

Table 9: Investigator Reporting Requirements

Report	Submit To	Description/Constraints
Withdrawal of IRB approval (either suspension or termination)	Sponsor	The investigator must report a withdrawal of approval by the reviewing IRB of the investigator's part of the investigation as soon as possible upon the center becoming aware.
Study Deviations	Sponsor and IRB	Any deviation from the clinical investigational plan shall be recorded together with the explanation of the deviation.<br>Reporting of study deviations should comply with IRB policies and/or local laws and must be reported to Medtronic as soon as possible upon the center becoming aware of the deviation.
Final Report	IRBs	This report must be submitted within 6 months of study completion or termination.

15.3 Sponsor Records

Medtronic shall maintain the following accurate, complete, and current records:

- All correspondence which pertains to the investigation
- Signed CTAs, current investigator curriculum vitae, delegated task list
- All signed and dated case report forms submitted by investigator, samples of informed consents, and other information provided to the subjects
- Copies of all IRB/ approval letters and relevant IRB correspondence
- Names of the institutions in which the clinical investigation will be conducted
- Correspondence with authorities as required by national legislation
- Forms for reporting any adverse events and adverse device effects
- Names/contact addresses of monitors
- Statistical analyses and underlying supporting data
- Final report of the clinical investigation
- The Clinical Investigation Plan, Investigator Brochure/Report of Prior Investigations, if required, and summary and study related reports
- Study training records for site personnel and Medtronic personnel involved in the study
- Forms for reporting any adverse events and adverse device effects.
- Any other records that local regulatory agencies require to be maintained.

15.4 Sponsor Reports

Medtronic shall prepare and submit the following complete, accurate, and timely reports listed in the tables below. In addition to the reports listed below, Medtronic shall, upon request of reviewing IRB, regulatory agency or FDA, provide accurate, complete and current information about any aspect of the investigation.

Medtronic safety data reporting requirements are listed in Section 8.2 and 8.3 of the Adverse Event section.

Table 10: Sponsor Reports

Report	Submit To	Description
Premature termination or suspension of the clinical investigation	Investigators, IRBs	Provide prompt notification of termination or suspension and reason(s).
Final report	Investigators, IRBs	A final report will be submitted to investigators and IRBs within six months after completion or termination of this study.
Study deviation	Investigators	Site specific study deviations will be submitted to investigators quarterly.

Medtronic records and reports will be stored in locked file cabinets at Medtronic during the course of the study. Electronic versions of the reports will be kept on a password-protected document management system. After closure of the study, all records and reports will be archived indefinitely.

Appendices

Appendix A: Case Report Forms

Below is a draft version of the e-CRF forms. Final e-CRFs will be provided to centers via the electronic data management system after the center has fulfilled all requirements for database access.

Baseline Form

**Administrative Information**	
Subject identifier for the study	____________
Date of baseline assessment	____/____/____
Date subject signed informed consent	____/____/____
Date subject signed privacy/protection authorization	____/____/____
**Demographics**	
Date of birth	____/____/____
Gender	O Male<br>O Female
Race/Ethnic origin	O Subject/physician chose not to provide information<br>O Not reportable per local laws or regulation<br>O American Indian or Alaska Native<br>O Asian<br>O Black or African American<br>O Hispanic or Latino<br>O Native Hawaiian or Pacific Islander<br>O White or Caucasian<br>O Two or more races<br>O Other Race, specify ____________
**Inclusion Criteria**	
**If any question is answered NO, then DO NOT include this subject in the study**	
Patient is indicated for continuous arrhythmia monitoring with an Implantable Cardiac Monitor	O No<br>O Yes
Patient is willing to undergo implant in clinic setting with only local anesthetic and/or oral anti-anxiety medications for sedation	O No<br>O Yes
Patient is 18 years of age or older	O No<br>O Yes
Patient is willing and able to provide consent and authorize the use and disclosure of health information	O No<br>O Yes

DRAFT

Patient is willing and able to comply with the protocol including the required Follow-up visit	O No<br>O Yes
**Exclusion Criteria**	
**If any question is answered YES, then DO NOT include this subject in the study**	
Patient has reduced immune function or is otherwise at high risk for infection	O No<br>O Yes
Patient has had a recent (within 30 days) or otherwise unresolved infection	O No<br>O Yes
Patient is implanted or indicated for implant with a pacemaker, ICD, CRT, or hemodynamic monitoring system	O No<br>O Yes
Patient is participating in another clinical study that may have an impact on the study endpoints	O No<br>O Yes
Patient's life expectancy is less than 1 year	O No<br>O Yes
Patient is pregnant	O No<br>O Yes
Patient has unusual thoracic anatomy or scarring at the implant site which may adversely affect the success of the implant procedure	O No<br>O Yes
**Testing Results**	
Height	____________<br>O cm<br>O in
Weight	____________<br>O kg<br>O lb
**Review of system**	
General cardiovascular history	□ NONE
	**Cardiomyopathy, specify**
	□ Cardiomyopathy, ischemic
	□ Cardiomyopathy, hypertropic
	□ Cardiomyopathy, restrictive
	□ Cardiomyopathy, dilated/congestive
	□ Cardiomyopathy, other specify<br>____________
General cardiovascular history *(continued)*	□ Congenital heart disease
	□ Congestive heart failure
	□ Coronary artery disease
	□ Idiopathic structural heart disease
	□ Hypertension
	□ Hypotension
	□ Primary / idiopathic electrical disease
	□ Pulmonary hypertension (PH)
	**Syncope, specify**
	□ Syncope, due to known VT
	□ Syncope, due to VF

DRAFT

DRAFT

	□ Syncope, idiopathic
	□ Syncope, due to carotid sinus
	□ Syncope, due to suspected VT
	□ Syncope, vasovagal
	□ Syncope, due to bradycardia
	□ Syncope, due to unknown etiology
Cardiovascular surgical history	□ NONE
	□ Abdominal aortic aneurysm repair
	□ Ablation
	**Ablation, specify location**
	□ Ablation, Atrial isthmus (right)
	□ Ablation, AV Node
	□ Ablation, epicardial
	□ Ablation, HIS bundle
	□ Ablation, MAZE
	□ Ablation, pulmonary vein
	□ Ablation, sinus node
	□ Ablation, unknown type
	□ Cardiac transplant
	□ Carotid endarterectomy
	□ Carotid stent
	□ Coronary artery bypass graft (CABG)
	**Coronary artery bypass graft (CABG), specify**
	□ Coronary artery bypass graft (CABG), 1 vessel
	□ Coronary artery bypass graft (CABG), 2 vessels
	□ Coronary artery bypass graft (CABG), 3 vessels
	□ Coronary artery bypass graft (CABG), 4 or more vessels
	□ Coronary artery bypass graft (CABG), unknown
	□ Coronary artery intervention
	□ Surgical MAZE
Spontaneous arrhythmia history *Do not include arrhythmias that are only induced during an EP Study. (check ALL that apply)*	
Atrial arrhythmias	□ NONE
	**Atrial fibrillation**
	□ Atrial fibrillation, paroxysmal
	□ Atrial fibrillation, persistent
	□ Atrial fibrillation, permanent
	□ Atrial flutter
	□ Atrial tachycardia
	□ AV node re-entrant tachycardia

	□ Premature atrial complexes
	□ Premature atrial complexes, non-conducted
	□ SA nodal re-entry
	□ Sinus node dysfunction
	□ Supraventricular tachycardia
	□ Other atrial arrhythmias, specify ____________________
Spontaneous arrhythmia history *(continued)*<br>*Do not include arrhythmias that are only induced during an EP Study.*<br>*(check ALL that apply)*	
Ventricular arrhythmias	□ NONE
	□ Long Q/T syndrome
	□ Ventricular fibrillation
	□ Ventricular flutter
	□ Ventricular tachycardia, non-sustained
	□ Ventricular tachycardia, sustained monomorphic
	□ Ventricular tachycardia, sustained polymorphic
	□ Ventricular tachycardia, sustained, unknown morphology
	□ Other ventricular arrhythmias, specify _________________
Spontaneous arrhythmia history *(continued)*<br>*Do not include arrhythmias that are only induced during an EP Study.*<br>*(check ALL that apply)*	
AV junctional arrhythmias and blocks	□ NONE
	□ 1st degree AV block
	□ 2nd degree AV block
	□ 3rd degree AV block
	□ AV junctional rhythm
	□ Left bundle branch block
	□ Intermediate bundle branch block
	□ Right bundle branch block
	□ Pre-excitation syndromes (e.g. Wolf Parkinson White)
	□ Premature junctional contractions
	□ Other AV junctional arrhythmias and blocks, specify ________________
Endocrine	□ NONE
	**Diabetes**
	□ Diabetes, type 1
	□ Diabetes, type 2
	□ Diabetes, gestational
	**Renal dysfunction**

	□ Renal dysfunction, requiring dialysis
	□ Renal dysfunction, not requiring dialysis
	□ Other endocrine, specify _______
Vascular	□ NONE
	□ Carotid artery disease
	□ Cerebrovascular accident (stroke)
	□ Deep vein thrombosis
	□ Transient ischemic attack
	□ Peripheral Edema
	□ Peripheral vascular disease
	□ Poor peripheral perfusion
	□ Pulmonary edema
	□ Pulmonary embolism
	□ Raynaud's phenomenon
	□ Vascular aneurysm
	□ Other vascular, specify _______
Other vascular, specify	____________________
Respiratory / pulmonary	□ NONE
	□ Sleep apnea
	□ Persistent cough
	□ Asthma
	□ Chronic obstructive pulmonary disease (COPD)
	□ Pneumonia
	□ Hemoptysis
	□ Other respiratory, specify ___________
Other relevant medical history, *specify any current diseases, conditions or symptoms*	__________________

DRAFT

Medication Form

Medications - Anticoagulants and Antiplatelets		
		Please complete one row per medication<br>OR<br>O Patient is not taking any Anticoagulant or Antiplatelet medications
	Start date	O On at baseline<br>O Same as implant<br>__/___/____
	Medication Name	______________________
	Was the medication titrated prior to the Reveal implant procedure?	O No<br>O Yes
	Stop date	__/___/____<br>O On at exit
	**New DCI**	
**Medications - Pain relievers, Antibiotics, Anti-anxiety and Local anesthetics**		
	Start date	O On at baseline<br>O Same as implant<br>__/___/____
	Medication Name	______________________
	Was the medication given due to the Reveal implant procedure?	O No<br>O Yes, complete Route and Time
	Route	O Intravascular<br>O Subcutaneous<br>O Intramuscular<br>O Oral (by mouth)<br>O Other, specify ________________
	Start Time	__:__ (24 hour clock)
	Stop date	__/___/____<br>O On at exit

DRAFT

Implant Form

**Administrative Information**	
Date of implant	__/___/____
Trained implanting physician	________________________
Primary reason for implant	O Diagnosis of unexplained syncope<br>O Mangement for known AT/AF<br>O Diagnosis of suspected AT/AF<br>O Other, specify ___________
**Pre-Implant preparation**	
Subject prep time - start	__:__ (24 hour clock)
Subject prep time - finish	__:__ (24 hour clock)
Subject temperature, oral	_____<br>O Not available
	O degrees F<br>O degrees C
Number of staff required for prep	_____ MD<br>_____ PA<br>_____ NP<br>_____ RN<br>_____ Tech<br>_____ Other, specify________
Was the subject prepped in the same room as implant?	O Yes - same room<br>O No - separate rooms
Was mapping performed?	O Yes<br>O No
If mapping was performed was the Vector Check mapping tool used?	O Yes<br>O No
Surgical site preparation (check all that apply)	□ None<br>□ Shaved with razor<br>□ Clipped<br>□ Cleansed, specify solution___________<br>□ Other, specify__________
Subject preparation (check all that apply)	□ None<br>□ Mask<br>□ Gown<br>□ Drape<br>□ Other, specify__________

DRAFT

Did the implanting physician prep in the same room as implant?	O Yes - same room<br>O No - separate rooms
Implant physician preparation (check all that apply)	□ Dry Scrub in the room<br>□ Wet scrub in the room<br>□ Wet scrub outside the room<br>□ Dry scrub outside the room<br>□ Mask<br>□ Gown<br>□ Single layer of gloves<br>□ Double layer of gloves<br>□ Other, specify___________
**System Implant**	
Subject implant time - start<br>(time of initial incision)	__:__ (24 hour clock)
Subject implant time - finish<br>(time of pocket closure)	__:__ (24 hour clock)
Number of staff required for implant	_____ MD<br>_____ PA<br>_____ NP<br>_____ RN<br>_____ Tech<br>_____ Other, specify_________
Record the size of the incision	O Same as device width (approximately 2cm)<br>O 1.5 times device width<br>O ≥ 2 times device width
How was bleeding controlled during the procedure? (check all that apply)	□ Not applicable<br>□ Cautery<br>□ Suture<br>□ Clamp<br>□ Gauze<br>□ Other, specify___________
Was the device implanted?	O Yes<br>O No, specify reason________
Device location	O 1 - between clavicle and 1st rib, *not recommended*<br>O 2 - between clavicle and 1st rib, *not recommended*<br>O 3 - between 1st and 4th rib<br>O 4 - between 1st and 4th rib<br>O 5 - inferior to 4th rib<br>O 6 - inferior to 4th rib<br>O Other, specify ____________

DRAFT

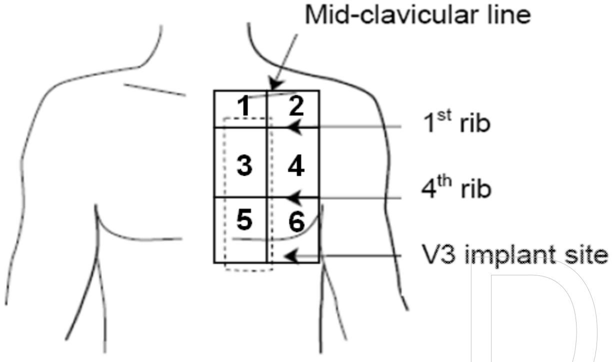	
Device orientation<br>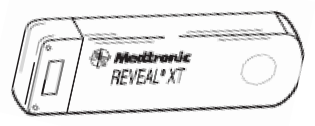<br>O Horizontal<br>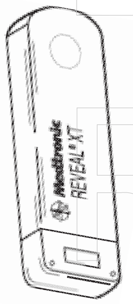<br>O Vertical<br>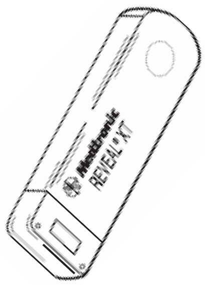<br>O 45 degrees<br>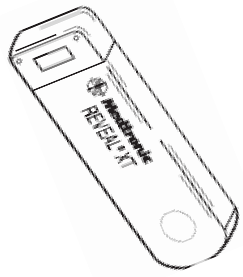<br>O 135 degrees<br>O Other, specify ___________	
Was the device placed as recommended with the electrodes outward (skin-facing)?	O Yes<br>O No
How many device locations were sutured?	O 0<br>O 1<br>O 2
Was the device pocket flushed?	O No<br>O Yes<br>O With antibiotics<br>O Without antibiotics
How many layers were sutured for wound closure?	O 1 layer<br>O 2 layer<br>O 3 layer
**Device information**	
Device model number	O Reveal DX model 9528<br>O Reveal XT model 9529
Device serial number	_______________
**Post Implant activities**	

DRAFT

Subject recovery time - start	__:__ (24 hour clock)
Subject recovery time - finish<br>(time patient left office)	__:__ (24 hour clock)
Number of staff required for recovery	_____ MD<br>_____ PA<br>_____ NP<br>_____ RN<br>_____ Tech<br>_____ Other, specify________
Did the subject recover in the same room as implant?	O Yes - same room<br>O No - separate rooms<br>O No recovery needed
R-wave amplitude	__.__ mV
**Physician survey**	
Did the patient tolerate the procedure in the office setting?	O Yes<br>O No - please describe__________
Please rate the speed of this implant procedure relative to a standard Reveal implant not done in-office	O Slower<br>O Similar<br>O Faster
Please rate the convenience for YOU (as implanting physician) conducting this procedure in-office relative to your standard facility for implanting Reveals	O Less convenient<br>O About the same convenience<br>O More convenient
Please rate your overall satisfaction with conducting this procedure in-office relative to a your standard facility for implanting Reveals	O Very satisfied<br>O Satisfied<br>O Neutral<br>O Dissatisfied<br>O Very dissatisfied
Was the room acceptable for the Reveal procedure?	O Yes<br>O No, specify______________
Did you have to modify your implant technique or other implant procedures compared to previous implants either hospital or office-based?	O No<br>O Yes, please describe_____________

DRAFT

Equipment Form

Please indicate which items were used for the typical Reveal Implant at your site. Even if you utilized the stock implant kit, please indicate the quantity of items that were utilized for the procedure, not necessarily all items tat were included in the kit.

**Procedure Items**	
**Prep**	
□ Sterile Gowns<br>□ Masks<br>□ Surgical Hats<br>□ Gloves (pairs)	Qty____<br>Qty____<br>Qty____<br>Qty____
□ CloroPrep<br>□ 4x4 Gauze pads<br>□ Sterile Towels<br>□ Other Prep, specify______	Qty____<br>Qty____<br>Qty____<br>Qty____
**Implant**	
□ 10cc syringe<br>□ Other syringe, specify______<br>□ Needle 25 gauge - 1.5"<br>□ Needle 27 gauge - .5"<br>□ Needle 22 gauge - 1"<br>□ Other Needle, specify_____	Qty____<br>Qty____<br>Qty____<br>Qty____<br>Qty____<br>Qty____
□ Scalpel #11<br>□ Scalpel #15<br>□ Other Scalpel, specify ____	Qty____<br>Qty____<br>Qty____
□ Saline flush<br>□ Disposable cautery - Bovie Battery powered<br>□ Other cautery, specify ____	Qty____<br>Qty____<br>Qty____
□ Tissue Forceps<br>□ Scissors<br>□ Needle Holder<br>□ Other Tool, specify_____	Qty____<br>Qty____<br>Qty____<br>Qty____
□ Silk Suture 3-0<br>□ Vicryl 2-0<br>□ Vicryl 3-0<br>□ Victryl 4-0<br>□ Other Suture, specify______	Qty____<br>Qty____<br>Qty____<br>Qty____<br>Qty____
**Dressings**	
□ 1/4" Steristrips<br>□ Mastisol/Dermabond<br>□ Thin Film Tagaderm Dressing 4x4<br>□ Thin Film Tagaderm Dressing 2x2<br>□ Non-Adherent Pad 3x4<br>□ Other Dressing, specify ____	Qty____<br>Qty____<br>Qty____<br>Qty____<br>Qty____<br>Qty____
**Other supplies**	

DRAFT

□ Small Drape fenistrated<br>□ Large Utility Drape<br>□ Sterile sleeve cover - for programmer head	Qty____<br>Qty____<br>Qty____
□ Other supply not listed above, specify_____<br>□ Other supply not listed above, specify_____<br>□ Other supply not listed above, specify_____<br>□ Other supply not listed above, specify_____<br>□ Other supply not listed above, specify_____	Qty____<br>Qty____<br>Qty____<br>Qty____<br>Qty____
**Room**	
Is a sink present in the procedure room?	O Yes<br>O No
Was the air flow controlled (filter or positive pressure)?	O Yes<br>O No
Please briefly describe the implant procedure room (i.e. standard exam room, modified exam room, echocardiography room, etc.)	Description ______________________________

DRAFT

30-Day Follow-up Form

Administrative Information		
	Date of visit	__/___/____
**Physical Exam**		
	After examination of the implant site, are there any signs of procedure related infections or other procedure related adverse events?	O No<br>O Yes, complete an Adverse Event form
	R-wave amplitude	__.__ mV

Reminder: Please upload device interrogations to Clinical Transfer

90-Day Follow-up Form

Administrative Information		
	Date of visit	__/___/____
	Type of visit	O Telephone contact<br>O Office visit
**Physical Exam**		
	After examination of the implant site, are there any signs of procedure related infections or other procedure related adverse events?	O No<br>O Yes, complete an Adverse Event form

Unscheduled Follow-up Form

Date of visit	__/___/____
Type of visit	O Telephone contact<br>O Office visit
Reason for visit	O Wound check<br>O Exit visit<br>O Other, specify________________
After examination of the implant site, are there any signs of procedure related infections or other procedure related adverse events?	O No<br>O Yes, complete an Adverse Event form
R-wave amplitude (only at exit visit)	__.__ mV

DRAFT

Technical Observation Form

Administrative Information	
Date of technical observation	__/___/____
**Description of Technical Information**	
Provide a detailed description of the observation and any action(s) taken due to the malfunction.	______________
Device model	________________________ (NA = Not Applicable)
Device serial number	________________________ (NA = Not Applicable)
Device lot / kit number	________________________ (NA = Not Applicable)

DRAFT

System Modification Form

**Administrative Information**	
Date of system modification	____/____/____
**Reason for System Modification**	
Specify reason(s) for system modification (check ALL that apply)	□ Pocket infection
	□ Device migration
	□ Battery depletion
	□ Other device implant (IPG, ICD, CRT)
	□ Diagnosis made
	□ Other reason, specify _______________
**System Modification information**	
**Device 1**	
Action taken	O Repositioned<br>O Explanted, replaced<br>O Explanted, not replaced
If repositioned or replaced, specify new device location (insert image 1)	O 1 - between clavicle and 1st rib, *not recommended*<br>O 2 - between clavicle and 1st rib, *not recommended*<br>O 3 - between 1st and 4th rib<br>O 4 - between 1st and 4th rib<br>O 5 - inferior to 4th rib<br>O 6 - inferior to 4th rib<br>O Other, specify ___________
If repositioned or replaced, specify new device location, Other, specify	_____________
If replaced, specify details of new device, manufacturer	LOV:<br>1 - Medtronic<br>2 - Biotronik<br>3 - Boston Scientific<br>4 - St. Jude<br>5 - Cardiac Control Systems<br>6 - Cook Vascular<br>7 - ELA Medical<br>8 - Enpath Medical<br>9 - Oscor Medical<br>10 - Osypka<br>11 - Possis<br>12 - Sorin Biomedica<br>13 - Vascor Medical<br>991 - Other
If replaced, specify details of new device, Other, specify	_____________

DRAFT

If replaced, specify details of new device, model	O Reveal DX model 9528<br>O Reveal XT model 9529<br>O Other, specify____________________
If replaced, specify details of new device, serial number	_____________
If explanted, was the component returned to Medtronic?	O No<br>O Yes
**Returned Product Information**	
For any explanted Medtronic component / device not returned to Medtronic, provide justification and final disposition of the component / device.	______________
Component should be returned in a Returned Product Kit.	
If a kit is needed, contact your local Medtronic representative	
Explanted devices should be returned to...	United States of America<br>Medtronic, Inc.<br>Product Performance Department T-172<br>7000 Central Ave. NE<br>Minneapolis, MN 55432, USA

DRAFT

Study Deviation Form

**Administrative Information**	
Date of deviation	____/____/____
Which visit is this deviation associated with?	O Baseline<br>O Implant<br>O 30-Day Follow-up<br>O 90-Day Follow-up<br>O System Modification<br>O Study exit<br>O Other
**Deviation**	
Describe the deviation	____________________
Reason for deviation	____________________

DRAFT

Study Exit Form

**Administrative Information**	
Date of study exit	____/____/____
**Reason for Study Exit**	
Please specify reason for study exit.	O Study completed<br>O Lost to follow-up<br>O Subject withdrew consent<br>**Subject requested withdrawal from the study**<br>O Subject relocated to another geographic location<br>O Subject requested withdrawal from the study, other, specify reason ____________________<br>**Investigator withdrew subject from the study**<br>O Investigator deems medically necessary, specify reason ________________<br>O Failure to maintain adequate study compliance<br>O Inclusion / exclusion criteria not met<br>O No implant attempted<br>O Device explant<br>O Investigator withdrew subject, other, specify reason ____________________<br>O Other, specify reason ____________________
Was the patient diagnosis achieved based on Reveal findings?	O Yes, specify date __/___/___<br>O No
**Lost to Follow-up Information**	
Only complete this section if the subject was lost to follow-up.	
Document the attempts to contact the subject.	
**First attempt**	
Date of attempted contact	____/____/____
Method of attempted contact	O Phone<br>O Written<br>O Other, specify _____________
**Second attempt**	
Date of attempted contact	____/____/____
Method of attempted contact	O Phone<br>O Written<br>O Other, specify _____________
**Third attempt**	
Date of attempted contact	____/____/____
Method of attempted contact	O Phone<br>O Written<br>O Other, specify _____________

DRAFT

Subject Death Form

**Death Information**	
Specify the primary cause of death	_______________
Write a detailed description of the subject's death including signs, symptoms, and treatments immediately leading up to the death	_______________
Death classification<br><br>**Cardiac death definition:** A death directly related to the electrical or mechanical dysfunction of the heart.	O **Sudden cardiac death:** Natural death due to cardiac causes, heralded by abrupt loss of consciousness within one hour of the onset of acute symptoms; preexisting heart disease may have been known to be present, but the time and mode of death are unexpected. If time of onset cannot be determined, SCD will aleternatively be defined as any unexpected cardiac death occurring out of the hospital or in the emergency room as dead on arrival.<br><br>O **Non-sudden cardiac death:** All cardiac deaths that are not classified as sudden cardiac deaths, including cardiac deaths of hospitalized subjects on inotropic support.<br><br>O **Non-cardiac death**: All deaths not classified as cardiac deaths<br><br>O Unknown classification
Location of subject at time of death	O Home<br>O Long-term care facility<br>O Hospital<br>O Unknown<br>O Other, specify_______________
**Specify the relatedness of the death to the Implant or System Modification procedure or the Device**	
Procedure	O Not related<br>O Related<br>O Unknown<br>Specify procedure<br>O Implant<br>O System modification
Device	O Not related<br>O Related<br>O Unknown

DRAFT

System Explant Information	
Indicate the system explant information. Return only Medtronic devices / components to Medtronic for analysis.	
Device explanted?	O No<br>O Yes, Medtronic component?<br>O No<br>O Yes, Sent to Medtronic?<br>O No<br>O Yes
For any explanted Medtronic component / device not returned to Medtronic, provide justification and final disposition of the component / device.	______________________
Component should be returned in a Returned Product Kit.	
If a kit is needed, contact your local Medtronic representative	
Explanted devices should be returned to...	United States of America<br>Medtronic, Inc.<br>Product Performance Department T-172<br>7000 Central Ave. NE<br>Minneapolis, MN 55432, USA
**Supporting Documentation**	
To ensure proper adjudication of this death by the Adverse Event Advisory Committee (AEAC), please send to Medtronic all supporting documentation such as:<br>- Death Summary<br>- Hospital discharge note(s) and/or hospital progress note(s) related to the death<br>- Device interrogation report /disk or printout, if performed<br>- Death certificate<br>- Autopsy report	
Was an autopsy performed?	O No<br>O Yes, *Submit autopsy report*<br>O Report submitted<br>O Not available at time of form completion
Was the device interrogated and saved to disk or printout?	O No, *explain below*<br>O Yes, *send disk or printout*
Explain why device was not interrogated	_________________________
**Reminders**	

DRAFT

- Perform a final device interrogation, if possible
- Document final disposition of any explanted system component on Device Disposition Log
- If explanted, send system to Product Performance Department in a Return Product Kit
- Complete the following forms:
  - Death Form
  - Adverse Event Form, to document the Adverse Event that led to the death
  - Adverse Event Update Form(s)

- Send the following to Medtronic, if available:
  - Death summary
  - Hospital discharge notes(s) and/or hospital progress note(s) related to the death
  - Death certificate
  - Autopsy report
  - Device interrogation report/ disk or printout, if performed

DRAFT

Adverse Event Form

**Administrative Information**	
Date of event onset	____/____/____
Specify time frame	O During implant<br>O Post-implant
Date study center became aware of the adverse event	____/____/____
**Description of Adverse Event**	
Identify the diagnosis:	______________
Describe the adverse event including relevant symptoms, clinical findings and underlying cause	______________
**Diagnostic Tests and Procedures**	
Indicate below all diagnostic tests / procedures supporting the diagnosis of this adverse event (check all that apply). Indicate, for each that is checked, whether the results were normal (negative) or **describe the abnormal findings that are relevant to the diagnosis of the adverse event.**	
□ Blood tests	
Test result	O All normal for the subject<br>O Abnormal, specify______________
□ Device interrogation	
Test result	O All normal for the subject<br>O Abnormal, specify______________
□ ECG	
Test result	O All normal for the subject<br>O Abnormal, specify______________
□ Echocardiogram	
Test result	O All normal for the subject<br>O Abnormal, specify______________
□ Chest X-ray	
Test result	O All normal for the subject<br>O Abnormal, specify______________
□ Cardiac stress testing	
Test result	O All normal for the subject<br>O Abnormal, specify______________
Cardiac stress testing, abnormal, specify results	____________________
□ Invasive imaging	

DRAFT

Specify tests and results	______________________
□ Other diagnostic tests / procedures	
Specify tests and results	______________________
**Action(s) Taken**	
Actions taken as a result of the adverse event (check ALL that apply)	
□ No action taken	
□ Inpatient Hospitalization	
Date of admission	____/____/____
Date of discharge	____/____/____<br>O Subject not discharged at time of form completion
Length of hospitalization	O Less than 24 hours<br>O 24 hours or more
□ Prolongation of existing hospitalization	
□ Emergency Department visit	
date:	____/____/____
□ Urgent Care visit	
date:	____/____/____
□ Clinic or office visit	
date:	____/____/____
□ Surgical/invasive procedure or treatment	
describe	______________________
Date	____/____/____
describe	______________________
Date	____/____/____
□ Device reprogramming (related to AE)	
describe	______________________
Date	____/____/____
□ Medications administered to treat this adverse event	
Medication name (generic)	______________________

DRAFT

Route	O Intravascular<br>O Subcutaneous<br>O Intramuscular<br>O Oral (by mouth)<br>O Other, specify ________________
Adjustments	O Initiated<br>O Discontinued<br>O Increased<br>O Decreased
□ Other actions taken	
describe	______________________
Date	____/____/____
describe	______________________
Date	____/____/____
**Specify the relatedness of the Adverse Event to the Implant or System Modification procedure**	
Procedure	O Not related<br>O Related<br>O Unknown<br>Specify procedure<br>O Implant<br>O System modification
**Regulatory Reporting**	
**Serious Adverse Event (SAE) definition (ISO 14155-1):**<br><br>**An Adverse Event that:**<br>**a) led to a death,**<br>**b) led to a serious deterioration in the health of the subject that**<br>**Resulted in a life-threatening illness or injury**<br>**Resulted in permanent impairment of a body structure or a body function**<br>**Required in-patient hospitalization or prolongation of existing hospitalization**<br>**Resulted in medical or surgical intervention to prevent permanent impairment to body structure or a body function**<br>**c) led to foetal distress, foetal death or a congenital abnormality or birth defect.**	
Is this adverse event a Serious Adverse Event (SAE)?	O No<br>O Yes
**Outcome**	

DRAFT

Indicate the outcome of the adverse event	O Resolved ____/____/____<br>O Unresolved, no further actions planned<br>O Unresolved, further actions or treatment planned (complete Adverse Event Update form as required)<br>O Unresolved at time of study exit / death / study closure<br>O Death Complete Death Form and submit death summary to Medtronic. (Check death only if death was a result of this adverse event.)
Indicate any outcome related comments you may have	________________

DRAFT

Adverse Event Update Form

Administrative Information	
Date of adverse event update	____/____/____
**Description of Adverse Event**	
Was there a change in the diagnosis since last reported to Medtronic?	O No<br>O Yes, specify________________
Yes, specify	______________
Describe the adverse event including relevant symptoms, clinical findings and underlying cause	______________
**Diagnostic Tests and Procedures**	
Indicate below all diagnostic tests / procedures supporting the diagnosis of this adverse event (check all that apply). Indicate, for each that is checked, whether the results were normal (negative) or **describe the abnormal findings that are relevant to the diagnosis of the adverse event.**	
Have there been any additional relevant diagnostic tests and procedures supporting the diagnosis of this adverse event?	O No<br>O Yes, indicate below for each that is checked whether the results were normal (negative) or describe the abnormal findings that are relevant to the diagnosis
□ Blood tests	
Test result	O All normal for the subject<br>O Abnormal, specify_____________
□ ECG	
□ Echocardiogram	
Test result	O All normal for the subject<br>O Abnormal, specify_____________
□ Cardiac stress testing	
Specify tests and results	_____________________
Specify tests and results	_____________________
□ No further action taken since last reported	
□ Inpatient Hospitalization	
Date of discharge	____/____/____<br>O Subject not discharged at time of form completion
Length of hospitalization	O Less than 24 hours<br>O 24 hours or more
□ Emergency Department visit	

DRAFT

date:	____/____/____
□ Urgent Care visit	
date:	____/____/____
□ Clinic or office visit	
date:	____/____/____
□ Surgical/invasive procedure or treatment	
describe	______________________
Date	____/____/____
describe	______________________
Date	____/____/____
□ Device reprogramming (related to AE)	
describe	______________________
Date	____/____/____
□ Medications administered to treat this adverse event	
Medication name (generic)	______________________
Route	O Intravascular<br>O Subcutaneous<br>O Intramuscular<br>O Oral (by mouth)<br>O Other, specify ________________
Adjustments	O Initiated<br>O Discontinued<br>O Increased<br>O Decreased
□ Other actions taken	
describe	______________________
Date	____/____/____
describe	______________________
Date	____/____/____
Was there a change in the relatedness of this adverse event?	O No<br>O Yes, complete for all procedures

DRAFT

Procedure	O Not related<br>O Related<br>O Unknown<br>Specify procedure<br>O Implant<br>O System modification
**Regulatory Reporting**	
**Serious Adverse Event (SAE) definition (ISO 14155-1):**<br><br>**An Adverse Event that:**<br>**a) led to a death,**<br>**b) led to a serious deterioration in the health of the subject that**<br>**Resulted in a life-threatening illness or injury**<br>**Resulted in permanent impairment of a body structure or a body function**<br>**Required in-patient hospitalization or prolongation of existing hospitalization**<br>**Resulted in medical or surgical intervention to prevent permanent impairment to body structure or a body function**<br>**c) led to foetal distress, foetal death or a congenital abnormality or birth defect.**	
Is this adverse event a Serious Adverse Event (SAE)?	O No<br>O Yes
**Outcome**	
Indicate the outcome of the adverse event	O Resolved ____/____/____<br>O Unresolved, no further actions planned<br>O Unresolved, further actions or treatment planned (complete Adverse Event Update form as required)<br>O Unresolved at time of study exit / death / study closure<br>O Death Complete Death Form and submit death summary to Medtronic. (Check death only if death was a result of this adverse event.)
Indicate any outcome related comments you may have	________________

DRAFT

Appendix B: Preliminary Publication Plan

Publications addressing the study data will be handled according to Medtronic Cardiac Rhythm Disease Management Standard Operating Procedures and as indicated in the Clinical Trial Agreement. This study will be registered per local regulatory requirements and on 'www.clinicaltrials.gov'. The publication strategy will be developed by the Publication Committee.

Publication Committee

Medtronic will form the Reveal In-Office Implants study Publication Committee (PC) that will manage publications utilizing data from this study with the goal of publishing results. The Publication Committee will develop the final Publication Plan as a separate document.

The Publication Committee's role is to guide publication development, review publications, and review publication ideas and proposals. In addition, the committee will apply and reinforce the authorship guidelines set forth in the Publication Plan. Membership in the Publication Committee does not guarantee authorship.

The study team will determine their criteria/approach for selecting PC members and a suitable PC chair from among the study investigators.

Management of Primary, Secondary and Ancillary Publications

The Publication Committee reviews all publications including primary, secondary and ancillary publications. Primary and secondary publications are those that address analyses of any or all primary objectives or secondary objectives, respectively, as specified in the Clinical Investigation Plan.

An ancillary clinical publication is any publication that does not address the study objectives identified in the Clinical Investigation Plan or Publication Plan and requires either Medtronic resources or data from a clinical study. It includes publications developed and sponsored by other Medtronic entities and may include but not be limited to Research, Reimbursement/Healthcare Economics personnel, and clinicians not participating in the clinical study.

The committee may decide that no publications/abstracts will be prepared prior to the end of the study or with individual center data.
Requests for publications utilizing regional data beyond the overall results will be evaluated for scientific validity and the ability of Medtronic to provide resources. The Publication Committee must approve publication of ancillary requests and will need to ensure that requests do not present conflicts with other geographical regions.

Criteria for Determining Authorship

Medtronic will adhere to the ICJME and journal specific authorship guidelines in selecting authors.

Authors, including Medtronic personnel, must at a minimum meet the conditions below:

- Substantial contribution to conception and design, or acquisition of data, or analysis and interpretation of data
- Drafting the article or revising it critically for important intellectual content
- Final approval of the version to be published

The Publication Committee will develop detailed authorship criteria as appropriate for the study. Demonstration of a high level of interest in the disease state, product and/or unique product feature being studied will be taken into consideration and combined with additional criteria to determine authorship order. The selected authors will be responsible for drafting the publication.

Appendix C: Data Monitoring Committee (DMC)

This study will not use a Data Monitoring Committee (DMC) to monitor the study

- There is low risk to subjects participating in the study
- All procedure related events will be reviewed by an independent AEAC and trending of the adverse events will continue throughout the study.
- The short enrollment period and low sample size makes a DMC impractical
- The short follow-up period makes it difficult for a DMC to meet and provide recommendations prior to the study ending
- The study is not randomized

Appendix D: Study Overview

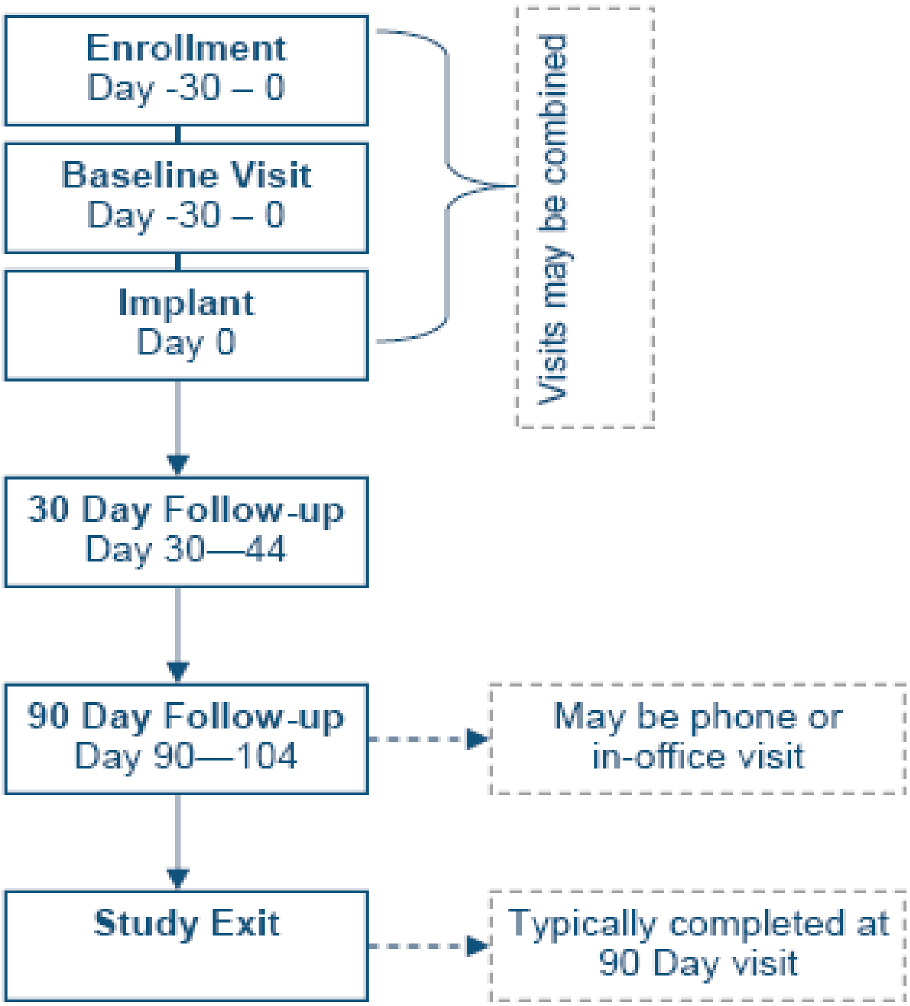

Appendix E: Informed Consent Template

Consent Form to Participate in a Research Study

SAMPLE

**TITLE:**	Reveal In-Office Implants Study
**PROTOCOL NO.:**	None
**SPONSOR:**	Medtronic, Inc.<br>Mounds View, Minnesota<br>United States
**INVESTIGATOR:**	Name<br>Address<br>City, State, Zip<br>Country
**SITE(S):**	Name<br>Address<br>City, State, Zip<br>Country
**STUDY-RELATED PHONE NUMBER(S):**	Name<br>Telephone Number

You are being asked to be in a research study involving human subjects. Research studies include only subjects who volunteer to participate. This consent form explains your rights and includes details about the study, such as its purpose, duration, required procedures, alternative treatments, and key contacts. The risks and potential benefits are also explained. You may withdraw from the study at any time. This consent form may contain words that you do not understand. Please ask the study doctor or the study staff to explain any words or information that you do not clearly understand. Carefully read the following and take time to discuss any questions that you may have with family, friends and the study team.

If you decide you want to be in this study, you will need to sign and date this consent form. You will receive a signed and dated copy of this consent form for your personal records, and you can refer to it while you are involved in this study.

Why is this research study being done? What is the purpose?

You are being asked to take part in this study because your study doctor believes you are at an increased risk for abnormal heart rhythms called cardiac arrhythmias. The U.S. Food and Drug Administration (FDA) has approved two device models called the

Reveal® DX and the Reveal® XT Insertable Cardiac Monitor ("ICM") devices (called the "Reveal ICM device" or "Reveal ICM devices" in this consent form) to monitor and assess heart rhythms. The Reveal ICM devices record heart activity and these recordings help doctors diagnose and decide how to treat abnormal heart rhythms. Because of this, your doctor is planning to implant a Reveal device under the skin of your chest to monitor your heart.

Typically, a Reveal ICM device is implanted in a hospital operating room or in a laboratory setting. However, the purpose of this study is to describe how the implant procedure is done in an office or clinic setting.

How long will I be in the study? How many other people will be in the study?

Participants in the study are referred to as subjects.

About 65 subjects will take part in this study in the United States; about 6 subjects will take part in the study at this site. Each subject in this study will participate for a minimum of 90 days. However, you may be in this research study for as long as 120 days depending on when your implant is scheduled.

What is involved in the research study?

SAMPLE

Study Procedures

If you decide to take part in this study, the study doctor and study nurse will collect information about you and your medical history, including any medications you take, and any other information in your medical records related to your condition or treatment that may be relevant to your participation in the study.

You will then have a minor surgery to insert one of the Reveal ICM devices below the surface of your skin in your chest region. Your study doctor will discuss the surgery with you and decide which Reveal ICM device is best for you. Your study doctor will then follow you according to the required study visits and may see you more often if medically necessary.

None of the procedures in this study are experimental.

Baseline In-office Visit

Your first study visit is an in-office visit with your study doctor or study nurse. This is called a baseline visit. During this visit, the following will be done:

- Information about your health and the medications you are currently taking will be collected.
- Your height and weight will be measured.

Reveal ICM Device Implant

Within 30 days (1 month) of signing this consent form and completing the Baseline Visit you will be implanted with one of the Reveal ICM devices. You may be asked to sign a separate consent form for this surgical procedure. During the implant procedure, a small incision will be made in your upper chest region and the device will be placed under your skin. You will be awake during the procedure; however, you may be given medication so you will not feel much, if any, discomfort.

During the implant procedure your study doctor or other members of the study team will collect information about the implant procedure including the materials used and the duration of the procedure.

The Reveal ICM device system includes the insertable cardiac monitor and the Patient Assistant. You may receive both of these components but only the insertable cardiac monitor will be used for this study. A description of each component is included below.

Reveal DX or Reveal XT Insertable Cardiac Monitor
The Reveal DX (model 9528) Insertable Cardiac monitor and the Reveal XT (model 9529) Insertable Cardiac monitor are small devices, about the size of a pack of chewing gum that are implanted under the skin in your chest. The devices continuously monitor your heart's natural rhythms and activity and can automatically detect and record abnormal heart rhythms. In addition, when using the Reveal Patient Assistant or Reveal XT Patient Assistant, you can activate the recording feature of the Reveal ICM devices when you are experiencing symptoms (see below).

Reveal Patient Assistant or Reveal XT Patient Assistant
The Reveal Patient Assistant (model 9538) and the Reveal XT Patient Assistant (model 9539) are hand-held, battery-operated devices. They enable you to record cardiac information in the Reveal ICM devices after experiencing symptoms of a possible cardiac event.

Trained Medtronic personnel may be present at the implant and at subsequent office visits and may use a device called a programmer to interact with your Reveal ICM device. The programmer is placed on your skin over the location of the Reveal ICM device. All of these activities will be done under the careful direction of your study doctor.

<u>30-Day In-office Follow-up Visit</u>
Thirty (30) days after your implant you will be scheduled for an in-office visit with your study doctor. At this study visit, your nurse or doctor will assess your health condition. In addition, the following procedures will be done:

- Your study doctor will examine the area of your chest where the device was inserted.
- Your Reveal ICM device will be checked using a Medtronic programmer to collect data stored in the device. This process is called a device interrogation.
- You will review your symptoms with your study doctor.

SAMPLE

- You will review your current medications list with your study doctor.
- You will discuss any unexpected events that have occurred since the implant. These events will be recorded and reported for study purposes.
- Your study doctor may run additional tests if they feel they are medically necessary.

90-Day In-office or Telephone Follow-up Visit
Ninety (90) days after your implant you will be scheduled for either an in-office or a telephone visit with your study doctor or nurse. At this study visit, your nurse or doctor will assess your health condition. In addition, the following procedures will be done:

- If your 90-day visit takes place in the office, your study doctor will examine the area of your chest where the device was inserted. If your 90-day visit takes place over the phone, you will be asked questions about the area of your chest where the device was inserted.
- You will review your symptoms with your study doctor or nurse.
- You will review your current medications list with your study doctor or nurse.
- You will discuss any unexpected events that have occurred since the implant. These events will be recorded and reported for study purposes.
- If your 90 day visit takes place in the office, your study doctor may run additional tests if they feel they are medically necessary.

Additional Visits
At any time during the course of the study, you or the study doctor may schedule extra in-office visits if the need arises. During these visits the study doctor will ask you for information about your health and what medications you are currently taking. Also, if you are seen in the hospital, emergency room, urgent care, or another clinic, please tell your study doctor as soon as possible as information about these visits may need to be reported as part of this study.

Study Exit
In most cases, your participation in the study will end on the day you have your 90-day in-office or telephone follow-up visit.

If your device needs to be surgically repositioned or removed at any time after the implant but before the end of the study, your participation in the study will end at that time.

If you have any unexpected events caused by the implant procedure, your participation in the study may continue until those events have been resolved.

When your participation in the study ends, you will be notified by your study doctor. At this time, you will be done with the study, but you will continue to receive standard of care treatment from your doctor.

Product Return

SAMPLE

The study doctor may remove the Reveal ICM device during the course of the study and send the explanted device back to Medtronic. Examples of why the study doctor may remove the Reveal ICM device are because of infection at the implant site, you need a different type of device, or the device has successfully diagnosed your condition and is no longer necessary. The study doctor may decide to implant another Reveal ICM device.

Whether or not you receive a new Reveal ICM device, your participation in the study will end and the study doctor will submit your health information to Medtronic.

If you die during the study, the study doctor will ask your family or other authorized representative for permission to remove the Reveal ICM device from your body. If the study doctor has this permission, the Reveal ICM device will be removed and sent back to Medtronic. Medtronic will collect information from the Reveal ICM device. Your family or other authorized representative does not have to give this permission.

If an autopsy is performed, a copy of the autopsy report and a copy of the death certificate, if available, will be sent to the Medtronic.

What are my responsibilities as a subject?

As a subject in a research study, you have certain responsibilities. You have the responsibility to be truthful regarding your health and medication history. You are expected to return to the study doctor's office for all in-office study visits and participate in the telephone visit (if applicable). If you are unable to participate in a scheduled visit, please call the study doctor's office to reschedule as soon as possible. You also have the responsibility to report any injuries, hospitalizations, emergency room visits, symptoms or complaints to the study doctor or study nurse as soon as possible.

What are the possible risks or discomforts?

The Reveal ICM devices have been approved for use by the U.S. Food and Drug Administration (FDA). Potential risks and discomforts associated with receiving a Reveal ICM device include:

- Rejection of the device by your body, which may have symptoms such as swelling, redness, or other irritation at or near the implant site
- Movement of the device from its initial implant location
- Infection at the site of the implant

It is not known if there are additional risks to you in having the Reveal ICM device implanted in an office setting instead of a hospital.

There may be other risks to you that are currently unknown.

Your condition may not get better or may become worse during this study.

SAMPLE

The risks to an embryo, fetus, or infant from exposure to the study device are unknown. Pregnant women are not allowed to participate in this study. If you suspect that you have become pregnant while participating in the study, tell the study doctor or study nurse as soon as possible. The study doctor will use his/her discretion to decide if you can continue participation.

What if new information becomes available about the study?

You will be told about anything new that is learned about this study and that may make you change your mind about staying in the study. You may be asked to sign a revised consent form if this occurs.

What are the possible benefits of the study?

If you agree to take part in this study, there may or may not be direct medical benefit to you. The information learned from this study may benefit future patients by improving implant procedures for the Reveal ICM device.

It is possible that having your implant in an office or clinic setting instead of in a hospital or laboratory will decrease the time required to prepare to have your implant surgery. Information collected from this study may assist in the design of new product(s)/procedures and/or instructions for use.

Who will provide funding?

Medtronic, Inc. is paying the study site for the work of collecting study data and managing the study at this site.

Will I have to pay for anything?

Medtronic will provide the Reveal ICM device at no cost to the study site, you or your insurer. Medtronic will also provide a fixed payment to the study site to cover the facility and physician charges related to the implant procedure. You will not have a co-payment, co-insurance, or deductible for the implant procedure. Medtronic will also compensate the study site for conducting the Baseline visit, the 30-day follow-up visit, and the 90 day follow-up visit.

All costs that are part of your usual medical care that you would have incurred regardless of your enrollment in this study, including the costs of any follow-up visits your doctors recommends, will be billed to your third party payer (your health insurer or Medicare). If your health insurer or Medicare requires any co-payment, co-insurance, or deductible, you will be responsible for making that payment. In some cases, there may be additional costs for which you will be billed.

SAMPLE

If you believe you have received a bill in error, please talk to the study doctor or study nurse.

If you have questions or concerns about your potential costs, please talk with your healthcare provider, your health insurer or Medicare.

Ask your study doctor to discuss the costs that will or will not be covered by the sponsor. Otherwise, you might have unexpected expenses from being in this study.

Will I be paid for being in this study?

You will not be paid for taking part in this study.

What other choices do I have if I do not participate?

You do not have to be in this study to be treated for a heart condition. You do not have to be in the study to receive a Reveal ICM device.

If you decide not to take part in this study, there is other care available to you. You may choose no treatment at all. You should discuss other treatments and their possible consequences with your physician.

What happens if I am injured or hurt during the study?

If the Reveal ICM device malfunctions, its standard warranty terms and conditions will apply. Medtronic has agreed to reimburse the study site for medical care it provides for any illness or injury caused by following the procedures required for the Study. Medtronic will not reimburse the study site if:

- The costs are covered by your health insurance or Medicare;
  - The illness or injury was caused by the negligence or intentional misconduct of the study staff or the study site;
- The study staff did not follow the protocol for the study;
  - The illness or injury is a result of the natural progression of your illness or Labels on the blood samples sent to the laboratory will not include individual patient names. your failure to follow your study doctor's directions.

To be eligible for reimbursement, the illness or injury must have occurred before the study closes at this site and the study site must notify Medtronic of the illness or injury within one year of the date the study closes at all study sites, or before the study closes at this site, whichever is earlier. If you believe that you have been injured as a result of your participation in the study, you should contact the study doctor."

Do I have the right to refuse to take part in the study or leave the study?

SAMPLE

Taking part in this study is voluntary. You may choose to not take part in the study or to leave the study at any time. If you choose not to participate in the study or to leave the study, it will not result in any penalty and you will not lose benefits to which you are entitled. Your regular care and your relationship with your doctors will not be affected.

You may leave the study simply by telling the study doctor. There are no consequences and no specific tests that are required before you leave the study, although it is requested that you complete your final study visit. All your health information already collected will be used.

The study doctors or the study sponsor may take you out of the study at any time without your permission if:

- The risks outweigh the benefits
- You do not follow the procedures requested by the study doctor or study nurse
- It is in your best interest
- Medtronic stops the study
- You do not consent to continue in the study after being told of changes in the research that may affect you;
- Or for any other reason.

If this happens, you will be told and the reasons will be explained to you.

How will Medtronic Use the Study Information?

If you decide to participate in the study, Medtronic, Inc. and others who work with the study will see health information about you.

The information collected about you may be used in several ways. Information about you and your health, that might identify you, may be given to others to carry out the research study. Your study doctor may use some of the information in making decisions about your research care. This will be described in the Authorization section of this consent form.

The sponsor of the study, Medtronic, Inc., may use the information in any of the following ways.

- To analyze and make conclusions about the results of the study
- In documents sent to government agencies throughout the world (for example, documents sent to the FDA to request approval of the device used in this study)
- For reporting side effects to the FDA and other government health agencies
- To provide overall study results, including your information, to other study doctors

SAMPLE

- To conduct new medical research, to reanalyze the study results in the future or to combine your information with information from other similar studies
- To develop new medical products and procedures, and other product-development related activities

While using the information in these ways, the sponsor may give it to its affiliated companies in the U.S. or other countries. The sponsor may also share the information with its business partners or companies it hires to provide study-related services. Information received during the study will not be used to market to you; your name will not be placed on any mailing lists or sold to anyone for marketing purposes.

Authorization to Use and Disclose Information for Research Purposes

This authorization section describes how your health information may be used and/or disclosed by your doctor (the study investigator), the hospital or clinic, and their respective staffs. The term "researchers and providers" will be used to include the group of people who may get personal information about you. These include the

- Study doctor
- Study staff
- Hospital or clinic (involved with a study procedure)
- Other health care providers involved in your care during the study.

Federal regulations give you certain rights related to your health information. These include the right to know who will be able to get the information and why they may be able to get it. The researchers and providers must get your authorization (permission) to use or give out any health information that might identify.

What information may be used and given to others?
If you choose to be in this study, the study doctor will get personal information about you from the researchers and providers. This may include information that might identify you. The study doctor may also get information about your health including:

- Past and present medical records
- Records about phone calls made as part of this research
- Records about your study visits
- Information obtained during this research about: Physical exams, questionnaires, laboratory and other test results
- Records about the study device

Who may use and give out information about you?
Information about your health may be used and given to the persons listed below by the researchers and providers. These persons might see the research information during and after the study.

SAMPLE

Who might get this information?
Your information may be given by the researchers and providers to the sponsor of this research, Medtronic, Inc. "Sponsor" includes any persons or companies that are working for or with the sponsor, or are owned by the sponsor.

Information about you and your health that are collected during the study, which might identify you may also be given by the researchers or the providers to:

- Other researchers participating in the study
- The U.S. Food and Drug Administration (FDA)
- Department of Health and Human Services (DHHS) agencies
- Other U.S. governmental agencies and governmental agencies in other countries
- Institutional review boards and other persons who are required to watch over the safety and effectiveness of medical products and therapies and the conduct of research.
- Other parties as required by law

Why will this information be used and/or given to others?
Information about you and your health that might identify you may be used and given to others to carry out the research study. The sponsor will analyze and evaluate the results of the study. In addition, people from the sponsor and its consultants may be visiting the research site. They will follow how the study is done, and they will be reviewing your information for this purpose.

The information may be given to the FDA. It may also be given to governmental agencies in other countries. This is done so the sponsor can receive marketing approval for new products resulting from this research. The information may also be used to meet the reporting requirements of governmental agencies.

The results of this research may be published in scientific journals or presented at medical meetings, but your identity will not be disclosed.

The information may be reviewed by institutional review or ethics boards who perform independent review of research as required by regulations.

What if I decide not to give permission to use and give out my health information?
By signing this consent form, you are giving permission to use and give out the health information listed above for the purposes described above. If you refuse to give permission, you will not be able to be in this research.

May I review or copy the information obtained from me or created about me?
You have the right to review and copy your health information in your medical record. However, if you decide to be in this study and sign this permission form, you will not be allowed to look at or copy your information until after the research is completed.

SAMPLE

May I withdraw or revoke (cancel) my permission?
Yes, but this permission does not have an expiration (ending) date.

You may withdraw or take away your permission to use and disclose your health information at any time. You do this by sending written notice to the study doctor. If you withdraw your permission, you will not be able to continue being in this study.

When you withdraw your permission, no new health information which might identify you will be gathered after that date. Information that has already been gathered may still be used and given to others as described in this form. For example, this would be done if it were necessary for the research to be reliable.

Is my health information protected after it has been given to others?
If you give permission to give your identifiable health information to a person or business, the information may no longer be protected. There is a risk that your information will be released to others without your permission.

Who can I call with questions, complaints or if I'm concerned about my rights as a research subject?

If you have any questions, concerns, or complaints about the research or your participation in this study, you should contact _________________ at ____________ .

If you think you have a research-related injury, you should contact _______________ at _________________.

If you have any questions about your rights as a research subject or if you have questions, concerns, or complaints about the research, you should contact:_________________ at _______________.

What does your signature on this consent form mean?

Your signature on this consent form means that:

- You accept the provisions of this consent form.
- You agree to join the study.

If you agree to be in this study, you will receive a signed and dated copy of this consent form.

Statement of the Subject:

SAMPLE

I have read this consent form and the research study has been explained to me. I have been given the opportunity to ask questions and have been told who to call if I have questions. No guarantee has been given to me about the possible results of the research study. My participation is voluntary, and I do not have to sign this consent form if I do not want to be part of this research study.

By signing this consent form, I am agreeing to be in the research study described in this consent form.

I authorize the use and disclosure of my health information to the parties listed in the authorization section of this consent for the purposes described above.

By signing this consent form, I have not given up any of my legal rights.

Printed Name of Subject____

CONSENT SIGNATURES:

Signature of Subject____

Date____
(must be written in by subject)

OR

Printed Name of Witness____

Signature of Witness____

Date____
(must be written in by witness)

Printed Name of Person Conducting
Informed Consent Discussion____

Signature of Person Conducting

SAMPLE

Informed Consent Discussion____

Date____
(must be written in by person conducting informed consent discussion)

SAMPLE

Appendix F: Participating Investigators and Institutions

At the time of Reveal In-Office Implants study Clinical Investigation Plan Version 1 completion, center activation was not finalized. A complete list of participating investigators and institutions where study activities will be conducted will be distributed under a separate cover when available.

Appendix G: IRB List

At the time of Reveal In-Office Implants study Clinical Investigation Plan Version 1 completion, center activation was not finalized. Therefore, a complete list of participating IRBs and the Chairperson(s) will be distributed under a separate cover when available.

Appendix H: Labeling

Labeling and reference/technical manuals for the Reveal DX and Reveal XT will be provided under separate cover. Labeling for all other market approved system components can be found with each device package insert.

Appendix I: Bibliography

1. Krahn AD, Klein GJ, Yee R, et al. Use of an extended monitoring strategy in patients with problematic syncope. Reveal Investigators. *Circulation.* 1999; 99:406–410.

2. Solano A, Menozzi C, Maggi R, et al. Incidence, diagnostic yield and safety of the implantable loop-recorder to detect the mechanism of syncope in patients with and without structural heart disease. Eur Heart J. 2004; 25(13):1116–9.

3. Krahn AD, Klein GJ, Yee R, et al. Detection of asymptomatic arrhythmias in unexplained syncope. Am Heart J. 2004 Aug;148(2):326-32.

4. Moya A, Brignole M, Menozzi C, et al. Mechanism of syncope in patients with isolated syncope and in patients with tilt-positive syncope. Circulation. 2001 Sep 11; 104(11):1261-7.

5. Hancox JG, Venkat AP, Coldiron B, Feldman SR, Williford PM. The Safety of Office-Based Surgery. Arch Dermatology 2004; 140:1379-1382.

6. Krahn AD, Klein GJ, Norris C, Yee R. The etiology of syncope in patients with negative tilt table and electrophysiological testing. Circulation.1995; 92:1819–24.

7. Krahn A.D., Klein G.J., Yee R., et al. Final results from a pilot study with an implantable loop recorder to determine the etiology of syncope in patients with negative noninvasive and invasive testing. American Journal of Cardiology (1998) 82:1 (117-119).

8. Seidl K, Breunung S, Rameken M, et al. Initial experience with an implantable loop recorder in patients with unexplained syncope. Z Kardiol. 2000 Jan; 89(1):43-50.

9. Mieszczanska H, Ibrahim B, Cohen TJ. Initial clinical experience with implantable loop recorders. J Invasive Cardiol. 2001 Dec; 13(12):802-4.

10. Krahn A.D., Klein G.J., Fitzpatrick A., et al. Predicting the outcome of patients with unexplained syncope undergoing pro-longed monitoring. PACE - Pacing and Clinical Electrophysiology (2002) 25:1 (37-41).

11. Vater M., Rameken M., Pitschner H.F., et al. The endless-loop-recorder in clinical practice - Results of the multicentre German Reveal® registry. Herzschrittmachertherapie und Elektrophysiologie (2002) 13:2 (101-109).

12. Armstrong V.L., Lawson J., Kamper A.M., et al. The use of an implantable loop recorder in the investigation of unexplained syncope in older people. Age and Ageing (2003) 32:2 (185-188).

13. Sanfins V, Chaves JC, Alves A, et al. Use of an implantable loop recorder: initial experience. Rev Port Cardiol. 2003 Dec;22(12):1479-83.

14. Mason P.K., Wood M.A., Reese D.B., et al. Usefulness of implantable loop recorders in office-based practice for evaluation of syncope in patients with and without structural heart disease. American Journal of Cardiology (2003) 92:9 (1127-1129).

15. Donateo P., Brignole M., Menozzi C., et al. Mechanism of syncope in patients with positive adenosine triphosphate tests. Journal of the American College of Cardiology (2003) 41:1 (93-98).

16. Ermis C., Zhu A.X., Pham S., et al. Comparison of automatic and patient-activated arrhythmia recordings by implantable loop recorders in the evaluation of syncope. American Journal of Cardiology (2003) 92:7 (815-819).

17. Paisey J.R., Yue A.M., Treacher K., et al. Implantable loop recorders detect tachyarrhythmias in symptomatic patients with negative electrophysiological studies. International Journal of Cardiology (2005) 98:1 (35-38).

18. Lombardi F, Calosso E, Mascioli G, et al. Utility of implantable loop recorder (Reveal Plus) in the diagnosis of unexplained syncope. Europace. 2005 Jan;7(1):19-24.

19. Inamdar V, Mehta S, Juang G, et al. The utility of implantable loop recorders for diagnosing unexplained syncope in 100 consecutive patients: five-year, single-center experience. J Invasive Cardiol. 2006 Jul; 18(7):313-5.

20. Maggi R, Menozzi C, Brignole M, et al. Cardioinhibitory carotid sinus hypersensitivity predicts an asystolic mechanism of spontaneous neurally mediated syncope. Europace. 2007 Aug; 9(8):563-7.

21. Brignole M, Menozzi C, Moya A, et al. Mechanism of syncope in patients with bundle branch block and negative electro-physiological test. Circulation. 2001 Oct 23; 104(17):2045-50.

22. Menozzi C, Brignole M, Garcia-Civera R, et al. Mechanism of syncope in patients with heart disease and negative electro-physiologic test. Circulation. 2002 Jun 11; 105 (23): 2741-5.

23. Pakarinen S, Oikarinen L, Toivaonen L, Short-term implantation-related complications of cardiac rhythm management device therapy: a retrospective single-centre 1-year survey. Europace 2010; 12:103-108

24. Wiegand U, LeJeune D, Boguschewski F, et al. Pocket Hematoma after pacemaker or implantable cardioverter defibrillator surgery. Chest 2004; 126(4): 1177-1186

25. Al-Khatib S, Lucas, F, Jollis J, et al. The relation between patients' outcomes and the volume of cardioverter-defibrillator implantation procedure performed by physicians treating Medicare beneficiaries. Heart Rhythm Disorders 2005; Oct 18; 46 (8):1536-40

26. Chamis A, Peterson G, Cabell C, et al. Staphylococcus aureus bacteremia in patients with permanent pacemakers or implantable cardioverter-defibrillators. Circulation. 2001; 104:1029-1033

27. Brignole M, Sutton R, Menozzi C, et al. Early application of an implantable loop recorder allows effective specific therapy in patients with recurrent suspected neurally mediated syncope. Eur Heart J. 2006; 27(9):1085–92.
